# Synthesis and biochemical characterization of naphthoquinone derivatives targeting bacterial histidine kinases

**DOI:** 10.1038/s41429-024-00726-2

**Published:** 2024-06-25

**Authors:** Teruhiko Ishikawa, Yoko Eguchi, Masayuki Igarashi, Toshihide Okajima, Kohei Mita, Yuri Yamasaki, Kaho Sumikura, Taisei Okumura, Yuna Tabuchi, Chigusa Hayashi, Martina Pasqua, Marco Coluccia, Gianni Prosseda, Bianca Colonna, Chie Kohayakawa, Akiyoshi Tani, Jun-ichi Haruta, Ryutaro Utsumi

**Affiliations:** 1 Graduate School of Education, Okayama University, Okayama, Japan; 2 Department of Science and Technology on Food Safety, Faculty of Biology-Oriented Science and Technology, Kindai University, Wakayama, Japan; 3 Institute of Microbial Chemistry (BIKAKEN), Tokyo, Japan; 4 SANKEN (The Institute of Scientific and Industrial Research), Osaka University, Osaka, Japan; 5 Istituto Pasteur Italy, Department of Biology and Biotechnology, “C. Darwin”, Sapienza University of Rome, Rome, Italy; 6 Department of Lead Exploration Units, Graduate School of Pharmaceutical Sciences, Osaka University, Osaka, Japan; 7 Compound Library Screening Center, Graduate School of Pharmaceutical Sciences, Osaka University, Osaka, Japan

**Keywords:** Small molecules, Drug discovery and development

## Abstract

Waldiomycin is an inhibitor of histidine kinases (HKs). Although most HK inhibitors target the ATP-binding region, waldiomycin binds to the intracellular dimerization domain (DHp domain) with its naphthoquinone moiety presumed to interact with the conserved H-box region. To further develop inhibitors targeting the H-box, various 2-aminonaphthoquinones with cyclic, aliphatic, or aromatic amino groups and naphtho [2,3-*d*] isoxazole-4,9-diones were synthesized. These compounds were tested for their inhibitory activity (IC_50_) against WalK, an essential HK for *Bacillus subtilis* growth, and their minimum inhibitory concentrations (MIC) against *B. subtilis*. As a result, 11 novel HK inhibitors were obtained as naphthoquinone derivatives (IC_50_: 12.6–305 µM, MIC: 0.5–128 µg ml^−1^). The effect of representative compounds on the expression of WalK/WalR regulated genes in *B. subtilis* was investigated. Four naphthoquinone derivatives induced the expression of *iseA* (formerly *yoeB*), whose expression is negatively regulated by the WalK/WalR system. This suggests that these compounds inhibit WalK in *B. subtilis* cells, resulting in antibacterial activity. Affinity selection/mass spectrometry analysis was performed to identify whether these naphthoquinone derivatives interact with WalK in a manner similar to waldiomycin. Three compounds were found to competitively inhibit the binding of waldiomycin to WalK, suggesting that they bind to the H-box region conserved in HKs and inhibit HK activity.

## Introduction

**Fig. 1:**
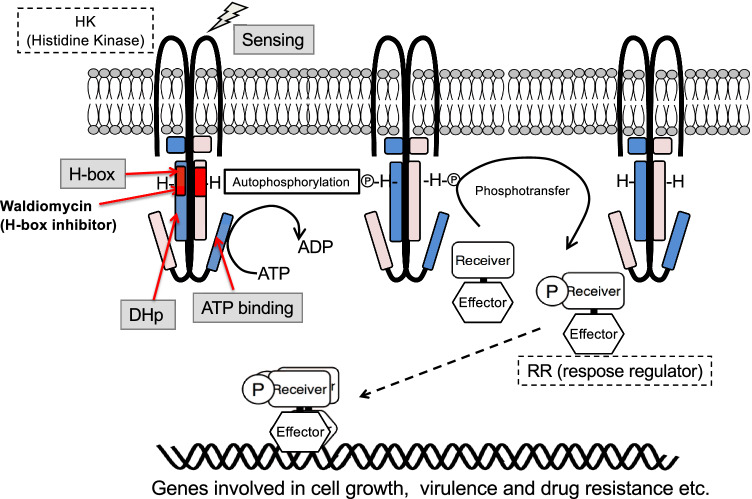
Bacterial two-component signal transduction (TCS)

Bacterial two-component systems (TCSs) form major pathways for signal transduction and are composed of a histidine kinase (HK), a membrane sensor, and a response regulator (RR), which functions mostly as a transcriptional regulator. In response to external environmental signals, the HK phosphorylates its own histidine residue (H) and transfers the phosphate group to the aspartate residue (D) in its associated RR. The phosphorylated RR then binds to target genes that are involved in cell division, pathogenicity, and drug resistance of pathogens, thereby controlling the expression of these genes (Fig. 1) [1, 2]. These systems are prevalent in bacteria, including hospital-acquired pathogens such as ESKAPE (*Enterococcus faecium, Staphylococcus aureus*, *Klebsiella pneumoniae, Acinetobacter baumannii*, *Pseudomonas aeruginosa, Enterobacter* species) [3], and are not found in the animal kingdom. Therefore, HK inhibitors have potential for development as next-generation agents against ESKAPE pathogens. These inhibitors could not only demonstrate antimicrobial activity but also exhibit antipathogenic and antidrug resistance properties [4].

Over the past 25 years, significant efforts have been made to discover various chemical series, including isothiazolidones, imidazolium salts, benzoxazines, salicylanilides, thiophenes, thiazolidones, benzimidazoles, and other derivatives. However, despite these endeavors, none of the identified HK inhibitors have progressed to clinical use, and none are currently undergoing clinical trials [5]. Previously reported HK inhibitors lack specificity to HK, and off-target effects against non-HK target proteins have been observed [6]. Furthermore, the structural information of the HK-compound complex remains undisclosed, posing a challenge to the development of HK-specific compounds.

**Fig. 2:**
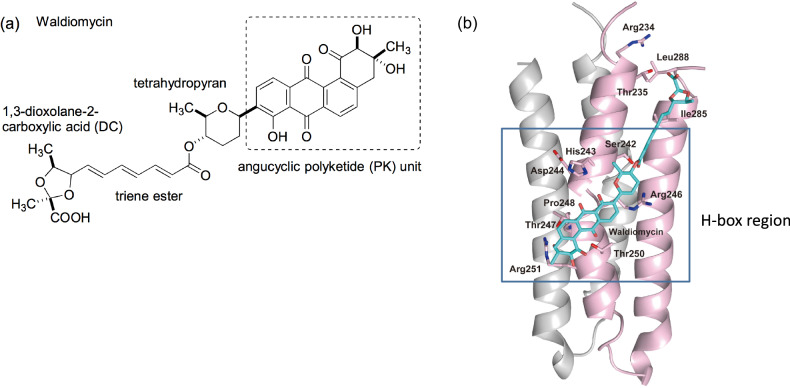
Structure of waldiomycin (**a**) and mode of waldiomycin bound H-box region of EnvZ DHp domain (**b**) [8]

Recently, we isolated a novel HK inhibitor, waldiomycin, from a *Streptomyces* extract (Fig. 2a) [7]. This inhibitor specifically inhibits a wide range of HKs, targeting the conserved H-box region of the intracellular dimerization domain (DHp: dimerization-inducing and histidine-containing phosphotransfer) of HKs. The naphthoquinone moiety of waldiomycin is believed to bind to the H-box region of the intracellular dimerization domain of HK (Fig. 2b) [8, 9]. This region is present in bacteria but not in mammalian cells, including humans [10]. It is postulated that waldiomycin blocks the access of the ATP-bound CA domain to the H-box. This unique mechanism of action establishes waldiomycin as the first known H-box inhibitor that impedes histidine phosphorylation in the H-box [8, 11]. Typically, kinase inhibitors target the ATP-binding site. To date, no other HK inhibitors targeting the H-box have been reported, aside from waldiomycin [5]. Naphthoquinone structures have demonstrated diverse biological activities, and extensive research has been conducted in the pharmaceutical, pesticidal, and other industrial fields. However, there have been no reports of these compounds inhibiting bacterial HKs [12–15]. The development of naphthoquinone derivatives as HK inhibitors holds great promise for broad-spectrum effects on HKs involved in bacterial growth, pathogenicity, and drug resistance.

In this study, we synthesized derivatives of naphthoquinone to enhance their affinity with the amino acid residues that make up the H-box in HK. We then evaluated the inhibitory potential of these derivatives against HK, specifically focusing on WalK, which is crucial for growth, as well as four other HKs involved in the virulence and drug resistance of pathogens.

## Materials and methods

### General

NMR spectra were recorded on a Varian 400MR or JEOL JNM-ECZ600R, with the solvent peak serving as the internal reference (CDCl_3_: *δ* H 7.26, *δ* C 77.0; DMSO-d_6_: *δ* H 2.50, *δ* C 39.5). High-resolution mass spectra were obtained using an Agilent Q-TOF G6520. Unless specified otherwise, materials were procured from commercial suppliers and used without further purification.

### Synthesis

**Scheme. 1:**
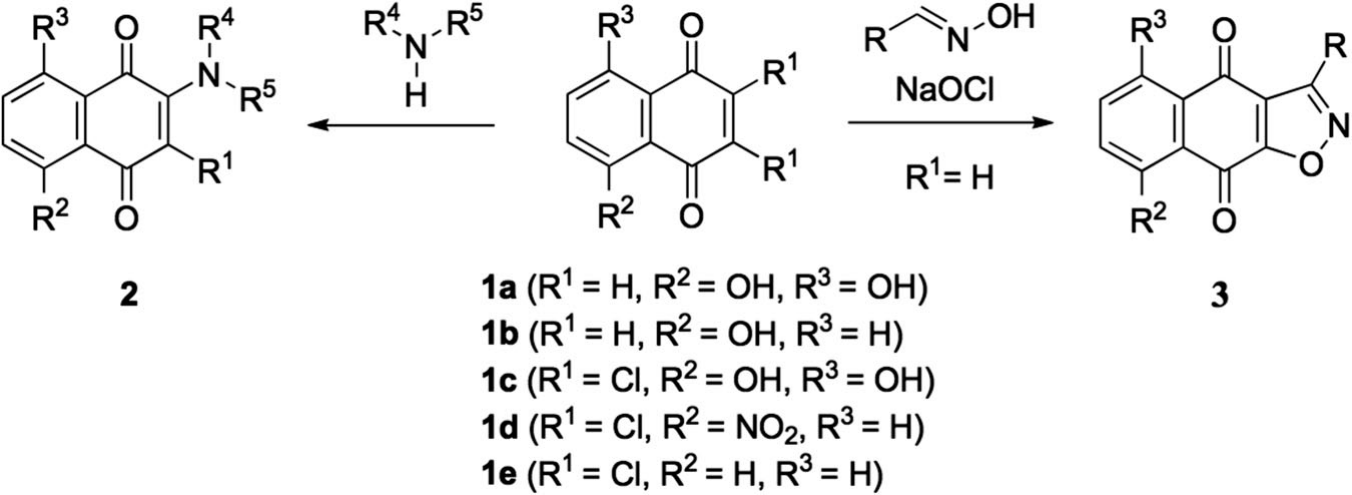
Synthetic methods for naphthoquinone derivatives

Waldiomycin is an angucycline antibiotic that consists of 1,3-dioxolane-2-carboxylic acid linked to an angucyclic polyketide via a triene ester linker and a tetrahydropyran (Fig. 2a) [7]. A complex model of waldiomycin and HK demonstrated that the functionalized anthraquinone backbone of waldiomycin, which contains an angucyclic polyketide (Fig. 2a), interacts with the H-box, which is a well-known conserved motif in the DHp domain of HKs including WalK and EnvZ (Fig. 2b) [8]. From this model, we hypothesized that naphthoquinones could serve as a basic framework for designing new HK inhibitors by imitating the modification of the angucyclic polyketide moiety. Various naphthoquinone derivatives can be synthesized from commercially available substrates through simple synthetic transformations, making them effective for constructing a chemical library for studying structure-activity relationships (Scheme 1).

Aminonaphthoquinones **2a**–**o** were synthesized through a Michael addition reaction using commercially available 1,4-naphthoquinones **1a**–**e** and corresponding amines, followed by further transformation if necessary. In the reactions, the naphthoquinone frameworks were constructed by air oxidation or elimination of chloride after the introduction of the amino groups to **1a**–**e**. Isoxazole-fused naphthoquinones **3a**–**c** were synthesized through a nitrile oxide [3 + 2] dipolar cycloaddition reaction using **1a** or **1b** and corresponding oximes, followed by oxidation reaction under the given conditions. Experimental procedures and characterization data for **2a**–**o** and **3a**–**c** are provided in the Supplementary Information.

### Cloning

To construct HK expression plasmids, regions encoding the cytoplasmic regions of PhoQ (261-486) and CpxA (188-457) were amplified through PCR using specific primers (Supplementary Information, Table S1), with the genomic DNA of the *Escherichia coli* strain MG1655 serving as the template and PrimeSTAR polymerase (Takara Bio). These amplicons were then digested with *Nde*I and *Xho*I and ligated into the pET22-b (+) vector (Novagen). The DNA sequences of the constructs were subsequently confirmed.

### Protein expression and purification

Expression plasmids (Supplementary Information, Table S2) were introduced into *E. coli* BL21(DE3) cells. The transformed cells were cultured in 2 × YT medium at 37 °C with aeration until they reached the mid-exponential phase, after which 0.1 mM IPTG was added for induction. Following a 5 h induction at 30 °C, the cells were harvested, washed with lysis buffer (50 mM Tris-HCl (pH 8), 100 mM NaCl), and stored at −80 °C until further use.

For the purification of His-tagged HKs, the frozen cells were resuspended in lysis buffer with 1 mM PMSF (phenylmethylsulfonyl fluoride, Nacalai tesque), lysed by sonication, and centrifuged at 17,800 × *g* for 10 min at 4 °C. For CpxA, protease inhibitor cocktail tablets (cOmplete^TM^ Ultra Mini, EDTA-free EASY pack protease inhibitor cocktail tablets, Roche) were added to the lysis buffer prior to sonication to prevent protein degradation. The supernatant was then affinity purified using Ni(II)-NTA agarose (Qiagen). The eluted protein samples were dialyzed against lysis buffer, concentrated using a centrifugal concentrator and stored at −20 °C with the addition of 30% glycerol.

### Inhibition assays of HK autophosphorylation

Autophosphorylation of WalK was conducted at 0.5 or 1 µM in a reaction mixture comprising 50 mM Tris-HCl (pH 8.5), 100 mM KCl, 5 mM MgCl_2_, 100 mM NH_4_Cl, 5% DMSO, and 40 µM ATP. The total reaction volume was 40 µl, and the incubation lasted for 30 min at 25 °C. To terminate the reaction, 40 µl of 2 × SDS-PAGE sample buffer was added, followed by a 10-min incubation at 37 °C. Subsequently, SDS-PAGE (10% acrylamide) was performed, and the samples were subjected to Western blotting using a PVDF membrane (Bio-Rad 1620177). The primary antibody used was anti-N3-phosphohistidine antibody (clone SC56-2) (EDM Millipore Corp), and the secondary antibody was goat anti-rabbit IgG-HRP (Abcam). A 2% bovine serum albumin (Sigma-Aldrich) solution served as the blocking reagent. Signals originating from phosphorylated HKs were detected using the HRP substrate (Immobilon Western Chemiluminescent HRP substrate, Millipore) and the Multi Imager II Multi Box (Bio Tools). Signal intensities were quantified with Image Analysis Software CS Analyzer 4 (ATTO). IC_50_ values (50% inhibitory concentrations) were determined using Prism 7 software (GraphPad).

In the assessment of naphthoquinone derivatives, the initial screening for inhibitory activity against WalK autophosphorylation was conducted at 50 or 100 µg ml^−1^. Compounds displaying inhibitory activity were further examined to ascertain their IC_50_ values against WalK. For the chosen compounds, inhibition against EvgS, EnvZ, PhoQ, and CpxA HKs was also assessed. These reactions involved the use of 1 µM HK, 40 µM ATP, and a 30 min incubation at 25 °C. Notably, autophosphorylation of 1 µM CpxA was carried out with 400 µM ATP, and that of 1 µM PhoQ was performed in a distinct reaction buffer consisting of 50 mM Tris-HCl (pH 7.5), 50 mM KCl, and 10 mM MgCl_2_.

### Extraction of total RNA and RT-qPCR

Total RNA was extracted from *B. subtilis* 168, which was cultured either in the presence or absence of naphthoquinone derivatives, following a previously described method with some modifications [16]. In brief, the cells were aerated and grown to an optical density at 660 nm of 0.3–0.4 at 37 °C. After the addition of DMSO or naphthoquinone derivatives, the cultures were further incubated for 5 min, cooled on ice for 1 min, and then harvested by centrifugation (2300 × *g*, 10 min, 4 °C). The cells were resuspended in 800 µl of RNA Lysis Buffer (SV Total RNA Isolation System, Z3100, Promega), transferred to a screw-capped tube containing 400 mg of glass beads (GB-01, TOMY), and disrupted using MicroSmash (MS-100, TOMY Medico) at 3000 rpm, 60 s ON/OFF for 6 cycles. RNA was then purified from 175 µl of the supernatant using the SV Total RNA Isolation System, followed by DNase I treatment (Turbo DNA-free^TM^ Kit, AM1907, Invitrogen) to remove any residual genomic DNA contamination. Reverse transcription reactions of 100 ng RNA samples were performed using the SuperScript^TM^ IV First-Strand Synthesis System (18091050, Invitrogen), and qPCR was conducted using the primers listed in Table S1 and TB Green Premix Ex Taq II (RR820A, Takara). The expression levels of the genes were normalized using the 16S rRNA gene as an internal standard. Each assay was performed at least three times. Statistical analyses were conducted using Tukey’s multiple comparison test (GraphPad Prism 7).

### Antimicrobial activity

The minimum inhibitory concentrations (MICs) were determined using the standard microtiter broth dilution method, as recommended by the guidelines of the Clinical Laboratory Standards Institute [17].

### Affinity selection/mass spectrometry

The affinity selection/mass spectrometry (AS/MS) is a method which can detect the binding of small compounds to proteins. This experiment was carried out following a previously established protocol [18] (Fig. 5), with the exception of using Millipore 96-well filter plates (MSBVN1210) instead of MAHAN4510. In the experiment, 1 µM of WalK (207-611, *B. subtilis*, refer to Table S2) was combined and incubated with 5 µM of waldiomycin and competitor compounds in a reaction buffer. The buffer composition was as follows: 25 mM Tris-HCl (pH 8.0), 50 mM KCl, 50 mM NH_4_Cl, 5 mM MgCl_2_, 1 mM DTT, and 0.1% CHAPS. Following the incubation, the mixture was subjected to size-exclusion chromatography via centrifugation to eliminate free compounds. The protein that was eluted was then denatured by adding an equal volume of methanol/0.1% formic acid. The compounds that were subsequently released from the protein were detected using an LC/MS system. The concentrations of waldiomycin were calculated from the value of peak areas using calibration curve that covered the range of 1 nM to 1 µM (*R*^2^ = 0.9991). The concentration of waldiomycin in the eluate of WalK and waldiomycin without competitor was 10.1 nM, and percentages to this value were calculated for other samples.

## Results and discussion

### WalK inhibitory activity and antibacterial activity of synthesized naphthoquinones

The inhibitory activities of naphthoquinones **2** and **3** on the HK (WalK) were evaluated and expressed as IC_50_ values. Upon analyzing the relationship between structure and HK inhibitory activity, several histidine kinase inhibitors were identified. These include 2-aminonaphthoquinones with a cyclic amino group (**2a**, **2b**, **2d**), an aromatic amino group (**2g**, **2h**, **2j**, **2l**, **2m**, **2o**), and naphtho[2,3-*d*]isoxazole-4,9-diones (**3a**, **3b**) (Table 1). Their IC_50_ values against WalK ranged from 12.6 to 305 µM, and their MICs against *B. subtilis* strain 168 ranged from 0.5 to 128 µg ml^−1^. Compounds **2c**, **2e**, **2f**, **2i**, **2k**, **2n**, and **3c** did not show any inhibitory activity against WalK. However, all naphthoquinone derivatives, except for **2k**, exhibited antibacterial activity.

**Table 1:** Structure, IC_50_, and MIC of naphthoquinone derivatives

Compounds	IC_50_ (μM)	MIC (μg ml^−1^)	Compounds	IC_50_ (μM)	MIC (μg ml^−1^)
	32.4	32		75.1	4
	48.4	8		N.I.	>128
	N.I.	8		13.0	8
	305	8		45.0	64
	N.I.	2		N.I.	128
	N.I.	8		25.9	16
	31.7	1		51.0	0.5
	12.6	8		186	0.5
	N.I.	4		N.I.	0.5
			Waldiomycin	13.1	8

IC_50_ values were determined against WalK from *B. subtilis*. MICs were determined against *B. subtilis* 168 strain grown in LB broth at 37 °C for 18 h

*N.I.* no inhibition

^a^Mixtures of regioisomers were used for biological assays

^b^Known in literature, see [14,15]

**Fig. 3:**
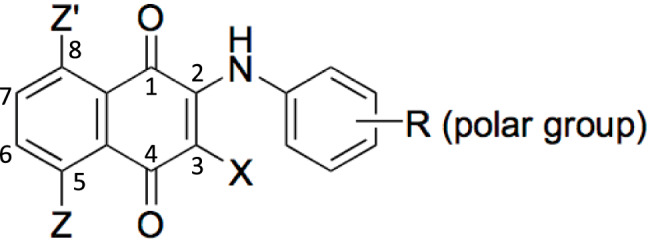
Prototype of H-box inhibitors. Z (OH or NH_2_, C5, C8) and R (polar groups) are essential for interacting with amino acid residues conserved in the H-box region

The structural features of naphthoquinone derivatives targeting WalK (HK) were found to be as follows: (1) The presence of hydroxyl groups on the 5,8-position of **2** or **3** was a crucial factor for inhibitory activity against WalK. Derivatives lacking hydroxyl groups (**2c** and **2i**) were inactive. (2) The effect of the amino group (**2o**) was confirmed, but the nitro group substituent (**2d** and **2n**) had no or only a weak effect. (3) Aliphatic amino derivatives (**2e** and **2f**) were inactive even if hydroxyl groups were introduced into the structure. (4) A noninhibitory compound such as **2k** has hydroxyl groups at both 5 and 8 positions, suggesting that the functional substituents on the aromatic amino group also affect the affinity and the inhibition ability. (5) The structure-activity relationship suggested that prototype structures as WalK (HK) inhibitors for 2-(arylamino)naphthoquinones **2** can be proposed (Fig. 3).

As shown in Table 1, correlation between WalK inhibitory activity and antimicrobial activity against *B. subtilis* was observed for some compounds. Compounds with WalK inhibitory activity comparable to waldiomycin (**2h** and **2l**) showed similar level of antimicrobial activity to waldiomycin. **2a**, **2m**, and **2o** showed weaker WalK inhibitory activity and antimicrobial activity. **2k** and **2n** did not inhibit WalK and showed no or a very weak antimicrobial activity. However, the other compounds with weaker or no WalK inhibitory activity exhibited antimicrobial activity equal to waldiomycin or even stronger (**2b**, **2c**, **2d**, **2e**, **2g**, **2f**, **2i**, **2j**, **3a**, **3b**, and **3c**). These compounds may exert antibacterial activity by interacting with proteins other than WalK, including other HKs and proteins involved in cell proliferation. Other factors may be the difference in permeability across the cell membrane and the accessibility to WalK. To further understand the nature of naphthoquinone derivatives that interact with WalK, a RT-qPCR (reverse transcription quantitative PCR) experiment was conducted.

### Effect against the expression of WalK/WalR regulated genes

**Fig. 4:**
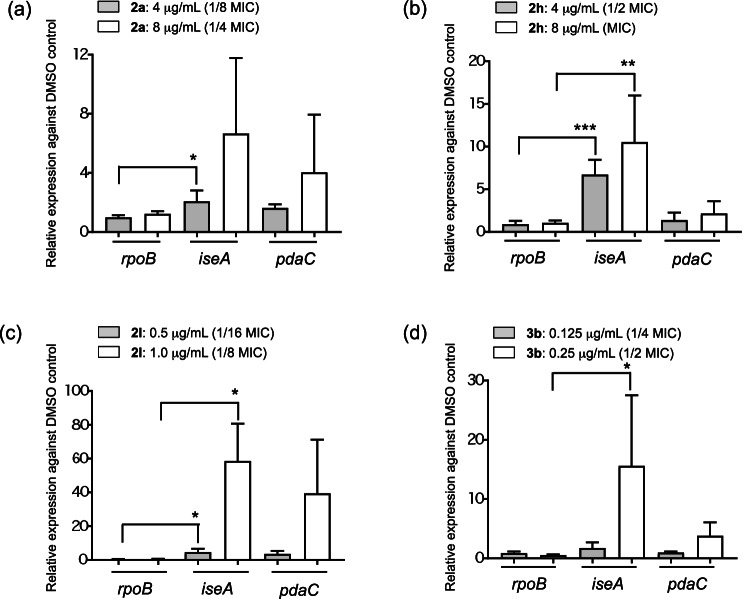
Expression of WalK/WalR down-regulated genes in *B. subtilis* cells after treatment with naphthoquinone derivatives. Mid-exponential phase cells were treated with subMIC or MIC doses of **2a** (**a**), **2h** (**b**), **2l** (**c**) and **3b** (**d**) for 5 min, their total RNA extracted, and analyzed by RT-qPCR. Expression of genes was normalized with that of 16S rRNA, and shown as relative expression against each DMSO control. Note the difference in the *y*-axis scale. Error bars represent the SD of at least three biological repeats. Statistical analyses were performed by Tukey’s multiple comparison test. *p < 0.05; **p < 0.01, ***p < 0.001

Among the naphthoquinone derivatives that demonstrated in vitro WalK inhibitory activity and antimicrobial effect against *B. subtilis* (Table 1), six compounds (**2a**, **2b**, **2h**, **2l**, **2o**, and **3b**) were chosen to verify if they also inhibited WalK in vivo, i.e., in *B. subtilis* cells. The WalK/WalR system in *B. subtilis* is active during the exponential growth phase of the cells [19]. Therefore, exponentially growing *B. subtilis* cells were treated with subMIC or MIC levels of the selected compounds. The changes in the expression of a WalK/WalR negatively regulated gene, *iseA* (formerly *yoeB*) [20], were analyzed using RT-qPCR. The *iseA* gene codes a negative regulator of D,L-endopeptidases and is associated with cell separation [21]. The expression of *rpoB* (a housekeeping gene) and *iseA* in each sample was normalized by the expression of 16S rRNA and was shown as relative expression against their DMSO treated control (Fig. 4). The expression of *iseA* significantly increased with the treatment of **2a**, **2h**, **2l**, and **3b** at subMIC or MIC levels, whereas the expression of *iseA* did not change with the treatment of **2b** and **2o** (data not shown). To confirm the inhibition of the WalK/WalR system, the changes in the expression of another WalK/WalR negatively regulated gene, *pdaC* (formerly *yjeA*), were also measured. This gene codes a polysaccharide deacetylase and is associated with lysozyme sensitivity [22]. As shown in Fig. 4, the expression of *pdaC* also tended to increase with the treatment of the four compounds, supporting the in vivo inhibition of WalK.

The results showed that **2a**, **2h**, **2l**, and **3b** significantly induced *iseA* gene expression, while **2b** and **2o** did not. These findings suggest that **2b** and **2o** may have other targets besides WalK in *B. subtilis* cells, which prevent them from binding to WalK.

### Affinity selection/mass spectrometry

**Fig. 5:**
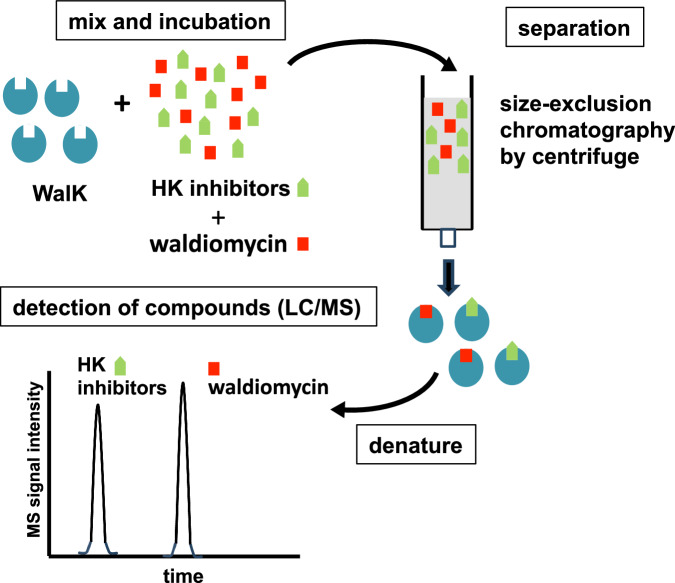
Principle of affinity selection/mass spectrometry

**Fig. 6:**
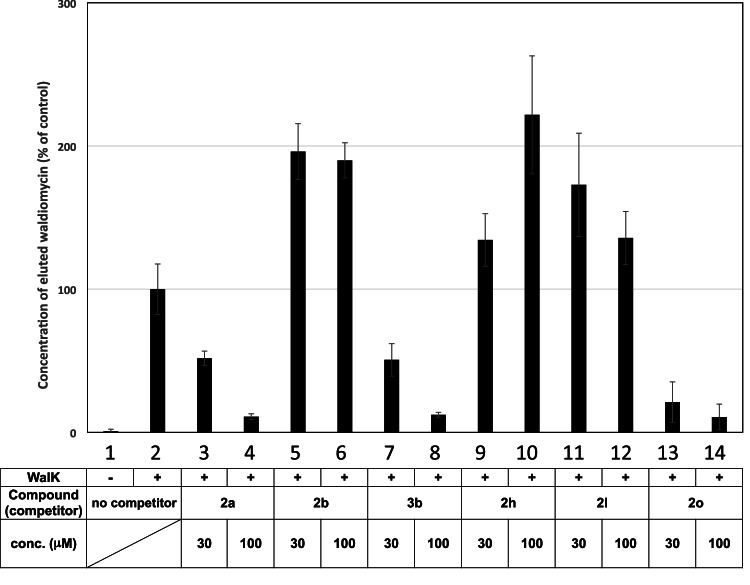
Affinity selection/mass spectrometry analysis. WalK (1 µM) was incubated with waldiomycin (5 µM, column 2) or with both waldiomycin (5 µM) and each compound (30 µM, columns 3, 5, 7, 9, 11, 13; 100 µM, columns 4, 6, 8, 10, 12, 14). In the absence of WalK, only waldiomycin (5 µM, column 1) was incubated. After mixing, AS/MS analysis was performed as described in “Materials and methods”. The value of peak area was expressed as relative values, with the value of waldiomycin and WalK protein as 100% (column 2). Data were the average of the quadruplicate results ± standard deviation

Inhibitors binding to a similar region show competitive binding. If the binding of naphthoquinone derivatives to WalK is competitive with that of waldiomycin, it will be a strong indication of those compounds binding to the H-box region. An affinity selection/mass spectrometry (AS/MS) binding analysis system was developed to evaluate the binding of waldiomycin to WalK (Fig. 5). Waldiomycin was quantitatively detected on a chromatogram by isolating the WalK-bound waldiomycin through size-exclusion chromatography from the mixture with WalK and applying it to LC/MS. However, a sample containing only waldiomycin without WalK did not yield a detectable signal (Fig. 6, column 1) because it eluted much slower than the protein. The effects of selected naphthoquinone derivatives on the binding of waldiomycin to WalK were further analyzed. The addition of **2a**, **2o**, or **3b** reduced the signal of waldiomycin in a dose-dependent manner (Fig. 6, columns 3, 4, 7, 8, 13, 14). Compared to the signal detected by mixing only waldiomycin with WalK (Fig. 6, column 2), only about 10% of the signal was observed in the presence of these compounds at 100 µM (Fig. 6, columns 4, 8, 14). These results suggest that the binding of **2a**, **2o**, or **3b** to WalK is competitive with that of waldiomycin, indicating that the binding sites of these compounds overlap with that of waldiomycin. In contrast, the addition of **2b**, **2h**, or **2l** increased the MS signal of the collected waldiomycin by about twofold compared to the signal in the presence of only waldiomycin (Fig. 6, columns 5, 6, 9, 10, 11, 12). These results suggest that the binding of **2b**, **2h**, or **2l** to WalK is not competitive with that of waldiomycin, but rather enhances the binding of waldiomycin.

A previous study showed that a single molecule of waldiomycin binds to an EnvZ dimer, even though the dimer potentially contains two H-box regions [8]. It is possible that the binding of waldiomycin to one chain of the EnvZ DHp region results in a conformational change in the other chain of the EnvZ dimer, inhibiting the binding of another waldiomycin molecule. Assuming that the WalK dimer similarly binds to a single molecule of waldiomycin, it is plausible that **2b**, **2h**, and **2l** may bind to the vicinity of the waldiomycin-binding site, thereby canceling the conformational change caused by waldiomycin. It is predicted that 100% level of the detected signal (Fig. 6, column 2) corresponds to one molecule of waldiomycin against the WalK dimer. Although this idea lacks sufficient experimental evidence, it well explains why **2b**, **2h**, and **2l** contribute to the binding of twice the amount of waldiomycin to WalK (Fig. 6, columns 5, 6, 9, 10, 11, 12). An ongoing structural biology study is expected to provide unambiguous evidence for this prediction.

### Naphthoquinone derivatives inhibiting HK activity of class I HKs

As previously reported [8], waldiomycin inhibited 13 class I HKs, including WalK, PhoQ, EnvZ, and CpxA. This broad-spectrum inhibition is attributed to waldiomycin binding to the H-box, which contains conserved amino acid residues found in the DHp domain of class I HKs. If the naphthoquinone derivatives exhibiting WalK inhibitory activity also target the conserved H-box region, they may inhibit the activity of other HKs. Therefore, we selected naphthoquinone derivatives to assess their inhibitory activity against other class I HKs, namely EvgS, EnvZ, PhoQ, and CpxA. These HKs play essential roles in the virulence and drug resistance of pathogens such as *Shigella, Salmonella, Klebsiella*, and *Pseudomonas* [2, 23, 24]. As indicated in Table 2, the chosen compounds, **2h**, **2l**, **2o**, and **3b**, inhibited all five HKs with IC_50_ values ranging from 0.1 to 186 µM. This implies that these compounds target a conserved region among HKs, similar to waldiomycin.

**Table 2:** IC_50_ of naphthoquinone derivatives targeting histidine kinases involved in virulence and drug resistance

HKs^a^			IC_50_ (μM)		
	Waldiomycin	2h	2l	2o	3b
EvgS^b^	66.8	25.8	50.0	0.20	109
EnvZ	33.1	47.2	26.8	1.30	77.3
PhoQ	10.9	42.3	133	0.10	96.3
CpxA	93.9	19.5	32.7	0.80	104
WalK	13.1	12.6	13.0	25.9	186

^a^HKs are from *Escherichia coli* K12

^b^EvgS (559–1197) D1009A: D1009A mutation was added to prevent the phosphor-relay from the autophosphorylated His901 to D1009

## Conclusions

In this study, we evaluated the inhibitory activity of *B. subtilis* WalK (HK) and the antibacterial activity against *B. subtilis* for the synthesized naphthoquinones. As a result, we analyzed six representative derivatives that exhibited both WalK inhibitory and antibacterial activities to clarify their interaction with WalK (Table 3). Mass spectrometry analysis was conducted to determine whether these naphthoquinone derivatives interacted with the H-box present in the DHp domain of WalK. The results showed that compounds **2a**, **2o**, and **3b** competitively interacted with waldiomycin and affected WalK (Fig. 6 and Table 3). Alternatively, **2b**, **2h**, and **2l** enhanced the binding of waldiomycin to WalK (Fig. 6). These results strongly suggest that compounds **2a**, **2o**, and **3b** interact with the H-box of WalK, similar to waldiomycin, while compounds **2b**, **2h**, and **2l** may facilitate the binding of waldiomycin to the H-box of WalK.

**Table 3:** Summary of biochemical characterization of naphthoquinone derivatives

Naphthoquinone	WalK	MIC (μg ml^−1^)	AS/MS	RT-qPCR	HKs
**2a**	+	+ (32)	+	+	NT
**2b**	+	+ (8)	−	−	NT
**2h**	+	+ (8)	−	+	+
**2l**	+	+ (8)	−	+	+
**2o**	+	+ (16)	+	−	+
**3b**	+	+ (0.5)	+	+	+
Waldiomycin [8, 9, 16]	+	+ (8)	NT	+	+

*WalK* + inhibitory effect against WalK, *MIC* + antibacterial activity against *Bacillus subtilis 168*, *AS/MS* + competitive inhibition of the binding of waldiomycin to WalK, − increased binding of waldiomycin to WalK, *RT-qPCR* + *iseA* gene expression was induced in *B. subtilis 168*, − no effect, *HKs* + inhibitory effect against HKs (EvgS, EnvZ, PhoQ, and CpxA), NT not tested

NMR analysis [8] has previously demonstrated that waldiomycin binds to the H-box in the DHp domain of EnvZ (HK) and is known to interact with class I HKs containing the H-box within their DHp domain. The synthesized WalK inhibitors in this study, namely **2h**, **2l**, **2o**, and **3b**, were assessed for their HK inhibitory activity or IC_50_ against EvgS, EnvZ, PhoQ, and CpxA, which are known to be involved in pathogenicity and drug resistance. The results indicated that these naphthoquinone derivatives from our study inhibited all four of these HKs in addition to WalK (Tables 2 and 3). Notably, when comparing the IC_50_ values of **2o** against WalK and its IC_50_ values against these four HKs, **2o** exhibited 100 times higher affinity for the nonWalK HKs.

This study is the first to reveal that compounds **2a**, **2o**, and **3b** competitively bind to the H-box in the HK’s DHp domain, antagonizing waldiomycin’s binding. This discovery suggests the potential synthesis of novel HK inhibitors with a new framework targeting the H-box, referred to as “H-box inhibitors,” extending beyond naphthoquinone derivatives.

### Supplementary information

Supplementary Information
