# Supplementary material for: Synthesis and biochemical characterization of naphthoquinone derivatives targeting bacterial histidine kinases
Source: J Antibiot (Tokyo). 2024 Jun 25;77(8):522–32. doi: 10.1038/s41429-024-00726-2 (PMC11284088; doi:10.1038/s41429-024-00726-2)
Supplement: Supplementary file 1 — Supplementary Information [file 41429_2024_726_MOESM1_ESM.pdf]

Synthesis and biochemical characterization of naphthoquinone derivatives targeting bacterial histidine kinases

Supplementary Information

Contents

1. Synthesis of naphthoquinone derivatives - 2

2. Primers used in this study (Table S1) - 27

3. Histidine kinases (HKs) used in the autophosphorylation assays (Table S2) - - - 28

1. Synthesis of naphthoquinone derivatives

General

NMR spectra were recorded on a Varian 400MR or JEOL JNM-ECZ600R, with the solvent peak serving as the internal reference ($CDCl_3$: δH 7.26, δC 77.0; DMSO-d_6: δH 2.50, δC 39.5). Multiplicities are denoted as s (singlet), d (doublet), t (triplet), q (quartet), m (multiplet), and br (broad). Coupling constants (*J*) are provided in Hertz (Hz). High-resolution mass spectra were obtained using an Agilent Q-TOF G6520. Unless specified otherwise, materials were procured from commercial suppliers and used without further purification.

Synthesis of 2-aminonaphthoquinones 2

Method A: Naphthoquinone **1** (0.1 mmol) and an amine (0.2 mmol) were dissolved in ethanol or acetonitrile (3 mL). The mixture was stirred for several hours at room temperature, or more elevated temperature if needed. The reaction progress was monitored on TLC analysis. The mixture was concentrated and the resulting residue was purified by column chromatography on silica gel using hexane–ethyl acetate or ethanol–ethyl acetate mixed solvent to give 2-aminonaphthoquinone **2**.

Method B: Naphthoquinone **1** (0.1 mmol), an amine (0.2 mmol), and triethylamine (0.2 mmol) were dissolved in ethanol or acetonitrile (3 mL). The mixture was stirred for several hours at room temperature, or more elevated temperature if needed. The reaction progress was monitored on TLC analysis. The mixture was concentrated and the resulting residue was purified by column chromatography on silica gel using hexane–ethyl acetate or ethanol–ethyl acetate mixed solvent to give 2-aminonaphthoquinone **2**.

The product structures, reaction conditions, and spectral data were listed below.

2-chloro-5,8-dihydroxy-3-(piperazin-1-yl)naphthalene-1,4-dione (**2a**): prepared by Method A (at room temperature for 12 hours in ethanol), 70 % yield.

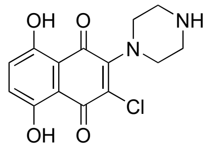

^{1}H NMR (400 MHz, $CDCl_3$) δ 2.05–2.15 (br, 1H), 3.02–3.08 (m, 4H), 3.56–3.64 (m, 4H), 7.18 and 7.23 (ABq, 2H, J = 9.4 Hz), 11.50-12.40 (br, 2H); ^{13}C NMR (150 MHz, $CDCl_3$) δ 46.8, 53.0, 110.5, 111.5, 122.7, 128.1, 130.1, 150.6, 156.7, 158.4, 181.5, 184.8; HRMS: ESI, m/z, $[M+H]^+$, Calcd. For $C_{14}H_{13}ClN_2O_4$: 309.0637, Found: 309.0635.

5-hydroxy-2-(piperazin-1-yl)naphthalene-1,4-dione (**2b**) : prepared by Method A (at room temperature for 12 hours in ethanol), 65 % yield.

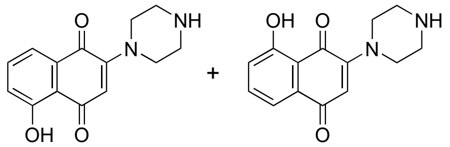

a mixture of regioisomers (1.1:1 ratio), ^{1}H NMR (400 MHz, $CDCl_3$): for major isomer δ 1.87–2.05 (br, 1H), 3.00–3.10 (m, 4H), 3.42–3.52 (m, 4H), 6.01 (s, 1H), 7.16–7.22 (m, 1H), 7.45– 7.60 (m, 2H), 12.63–12.77 (br, 1H); for minor isomer δ 1.87–2.05 (br, 1H), 3.00–3.10 (m, 4H), 3.52–3.58 (m, 4H), 5.90 (s, 1H), 7.16–7.22 (m, 1H), 7.45– 7.60 (m, 2H), 11.82–11.98 (br, 1H); ^{13}C NMR (100 MHz, $CDCl_3$) for regioisomer mixture: δ 42.9, 43.0, 50.2, 50.4, 109.5, 113.8, 114.5, 115.8, 118.2, 119.4, 123.2, 124.3, 132.3, 132.9, 134.2, 137.0, 153.4, 154.5, 160.5, 162.0, 182.5, 183.0, 187.8, 188.9; HRMS: ESI, m/z, $[M+H]^+$, Calcd. For $C_{14}H_{14}N_2O_3$: 259.1077, Found: 259.1074.

2-chloro-3-(piperazin-1-yl)naphthalene-1,4-dione (**2c**) : prepared by Method A (at room temperature for 12 hours in ethanol), 72 % yield.

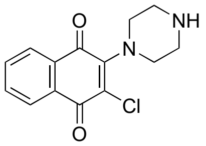

^{1}H NMR (400 MHz, $CDCl_3$) δ 2.05–2.20 (br, 1H), 3.02–3.07 (m, 4H), 3.52–3.60 (m, 4H), 7.61–7.73 (m, 2H), 7.97–8.01 (m, 1H), 8.10–8.15 (m, 1H); ^{13}C NMR (100 MHz, $CDCl_3$) δ 46.7, 52.7, 122.8, 126.5, 126.8, 131.4, 131.5, 133.0, 134.0, 150.0, 178.0, 181.8; HRMS: ESI, m/z, $[M+H]^+$, Calcd. For $C_{14}H_{13}ClN_2O_2$: 277.0738, Found: 277.0745.

3-chloro-5-nitro-2-(piperazin-1-yl)naphthalene-1,4-dione (**2d**) : prepared by Method A (at room temperature for 5 hours in ethanol), 61 % yield.

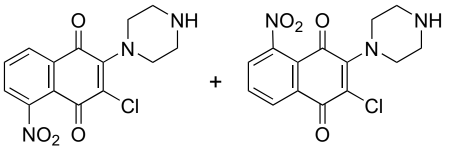

a mixture of regioisomers (1.1:1 ratio), ^{1}H NMR (400 MHz, $CDCl_3$): for major isomer δ 1.70–1.90 (br, 1H), 3.01–3.10 (m, 4H), 3.56–3.65 (m, 4H), 7.75–7.83 (m, 2H), 8.35 (dd, 1H, J = 2.2, 7.0 Hz); for minor isomer δ 1.72–1.90 (br, 1H), 3.01–3.10 (m, 4H), 3.56–3.65 (m, 4H), 7.62 (dd, 1H, J = 1.2, 7.7 Hz), 7.75–7.83 (m, 1H), 8.20 (dd, 1H, J = 1.2, 7.8 Hz); ^{13}C NMR (100 MHz, $CDCl_3$) for regioisomer mixture: δ 46.7, 52.8, 53.0, 119.2, 122.4, 122.7, 125.2, 127.0, 127.6, 129.1, 129.4, 132.3, 132.6, 133.6, 134.1, 148.3, 148.4, 149.6, 150.6, 173.9, 175.8, 179.1, 180.0; HRMS: ESI, m/z, $[M+H]^+$, Calcd. For $C_{14}H_{12}ClN_3O_4$: 322.0589, Found: 322.0598.

2-chloro-5,8-dihydroxy-3-((2-hydroxyethyl)amino)naphthalene-1,4-dione (**2e**): prepared by Method A (at room temperature for 5 hours in ethanol), 54 % yield.

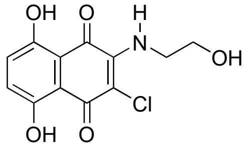

^{1}H NMR (400 MHz, $CDCl_3$) δ 1.58–1.80 (br, 1H), 3.82–3.98 (m, 2H), 4.06–4.18 (m, 2H), 6.60–6.70 (br, 1H), 7.12 and 7. 28 (ABq, 2H, J = 9.4 Hz), 11.83 (s, 1H), 12.96 (s, 1H); ^{13}C NMR (150 MHz, $CDCl_3$) δ 46.5, 61.7, 110.1, 110.4, 126.9, 131.1, 131.5, 145.0, 156.5, 158.4, 180.8, 182.1; HRMS: ESI, m/z, $[M–H]^–$, Calcd. For $C_{12}H_{10}ClNO_5$: 282.0175, Found: 282.0172.

2-chloro-3-((3,4-dihydroxyphenethyl)amino)-5,8-dihydroxynaphthalene-1,4-dione (**2f**): prepared by Method A (at room temperature for 12 hours in ethanol), 18 % yield.

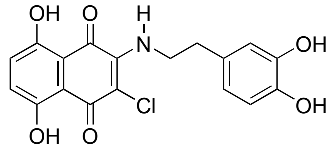

^{1}H NMR (400 MHz, DMSO-d_6) δ 2.68–2.78 (m, 2H), 3.84–3.99 (m, 2H), 6.48 (d, 1H, J = 7.6 Hz), 6.62 (s, 1H), 6.63 (d, 1H, J = 7.6 Hz), 7.20 and 7.35 (ABq, 2H, J = 9.4 Hz), 7.70–7.90 (br, 1H), 8.60–8.95 (br, 2H), 11.50–11.63 (br, 1H), 13.11 (brs, 1H); ^{13}C NMR (150 MHz, DMSO-d_6) δ 36.5, 45.4, 110.3, 110.9, 115.6, 115.8, 116.1, 119.4, 126.6, 129.1, 130.5, 143.9, 145.3, 155.0, 156.6, 180.1, 181.6; HRMS: ESI, m/z, $[M–H]^–$, Calcd. For $C_{18}H_{14}ClNO_6$: 374.0437, Found: 374.0439.

2-chloro-5,8-dihydroxy-3-((3-hydroxyphenyl)amino)naphthalene-1,4-dione (**2g**): prepared by Method B (at room temperature for 12 hours in ethanol), 51 % yield.

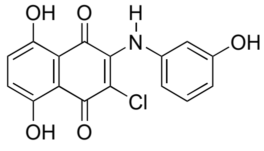

^{1}H NMR (400 MHz, $CDCl_3$) δ 4.96 (s, 1H), 6.59 (t, 1H, J = 2.4 Hz), 6.64–6.76 (m, 2H), 7.21 and 7.34 (ABq, 2H, J = 9.4 Hz), 7.22 (t, 1H, J = 8.0 Hz), 7.78 (brs, 1H), 11.87 (s, 1H), 12.82 (s, 1H); ^{13}C NMR (150 MHz, DMSO-d_6) δ 110.7, 111.1, 111.6, 112.3, 115.6, 127.3, 128.6, 130.1, 139.3, 143.9, 155.3, 156.5, 157.1, 181.1, 182.3; HRMS: ESI, m/z, $[M–H]^–$, Calcd. For $C_{16}H_{10}ClNO_5$: 330.0175, Found: 330.0176.

2-chloro-3-((4-chloro-3-hydroxyphenyl)amino)-5,8-dihydroxynaphthalene-1,4-dione (**2h**): prepared by Method B (at room temperature for 12 hours in ethanol), 44 % yield.

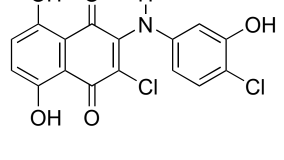

^{1}H NMR (400 MHz, $CDCl_3$) δ 3.72 (brs, 2H), 6.25 (d, 1H, J = 2.5 Hz), 6.43 (dd, 1H, J = 2.5, 8.6 Hz), 7.18 (d,

1H, J = 8.6 Hz), 7.28 and 7.33 (ABq, 2H, J = 9.4 Hz), 12.01 (s, 1H), 12.42 (s, 1H); ^{13}C NMR (150 MHz, DMSO-d_6) δ 104.5, 110.3, 110.5, 112.1, 112.2, 130.3, 130.8, 131.1, 131.9, 146.4, 152.5, 153.9, 159.3, 159.7, 179.1, 180.6; HRMS: ESI, m/z, [M-H]$^-$, Calcd. For $C_{16}H_9Cl_2NO_5$: 363.9785, Found: 363.9790.

2-chloro-3-((4-chloro-3-hydroxyphenyl)amino)-5,8-dihydroxynaphthalene-1,4-dione (**2i**): prepared by Method B (at room temperature for 12 hours in ethanol), 67 % yield.

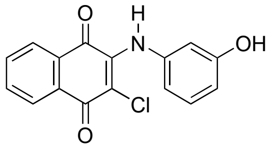

^{1}H and ^{13}C NMR data were identical for literature.[1]

HRMS: ESI, m/z, [M+H]$^+$, Calcd. For $C_{16}H_{10}ClNO_3$: 300.0422, Found: 300.0423.

2-((4-chloro-3-hydroxyphenyl)amino)-5-hydroxynaphthalene-1,4-dione (**2j**): prepared by Method A (at room temperature for 12 hours in ethanol), 21 % yield.

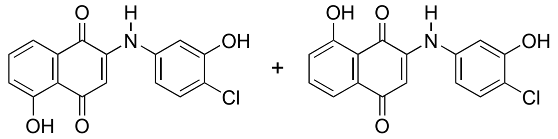

a mixture of regioisomers (1.1:1 ratio), ^{1}H NMR (400 MHz, DMSO-d_6) for major isomer: δ 6.08 (s, 1H), 6.84 (d, 1H, J = 8.2 Hz), 7.02 (s, 1H), 7.30–7.50 (m, 2H), 7.58–7.83 (m, 2H), 9.56 (s,1H), 10.48 (s, 1H), 11.53 (s, 1H); for minor isomer: δ 6.12 (s, 1H), 6.81 (d, 1H, J = 8.2 Hz), 7.01 (s, 1H), 7.30–7.50 (m, 2H), 7.58–7.83 (m, 2H), 9.90 (s,1H), 10.42 (s, 1H), 11.58 (s, 1H); ^{13}C NMR (150 MHz, DMSO-d_6) for regioisomer mixture: δ 101.2, 102.9, 108.2, 111.3, 111.6, 112.6, 114.2, 114.3, 115.5, 116.5, 117.7, 118.8, 124.6, 124.7, 125.0, 125.9, 130.3, 130.6, 134.8, 137.4, 137.6, 138.5, 145.9, 147.1, 153.0, 153.5, 160.0, 160.5, 182.1, 183.5, 186.5, 187.1; HRMS: ESI, m/z, [M–H]$^-$, Calcd. For $C_{16}H_{10}ClNO_4$: 314.0226, Found: 314.0224.

2**-chloro-5,8-dihydroxy-3-((4-methoxyphenyl)amino)naphthalene-1,4-dione** (**2k**): prepared by Method B (at room temperature for 12 hours in ethanol), 22 % yield.

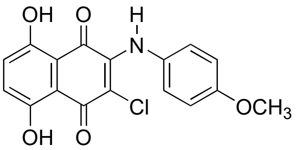

^{1}H NMR (400 MHz, $CDCl_3$) δ 3.83 (s, 3H), 6.88 (d, 2H, J = 8.8 Hz), 7.06 (d, 2H, J = 8.8 Hz), 7.18 and 7.30 (ABq, 2H, J = 9.4 Hz), 7.80 (bs, 1H), 11.84 (brs, 1H), 12.91 (s, 1H); ^{13}C NMR (150 MHz, $CDCl_3$) δ 55.5, 110.3, 110.5, 112.3, 113.6, 126.5, 127.3, 129.7, 131.6, 142.4, 156.8, 158.1, 158.4, 181.3, 182.2; HRMS: ESI, m/z, [M–H]$^-$, Calcd. For $C_{17}H_{12}ClNO_5$: 344.0331, Found: 344.0332.

tert-butyl (3-((3-chloro-5,8-dihydroxy-1,4-dioxo-1,4-dihydronaphthalen-2-yl)amino)phenyl)carbamate (2l): prepared by Method B (at 50 °C for 12 hours in acetonitrile), 55 % yield.

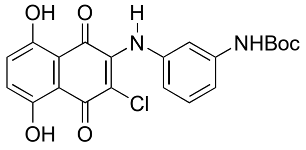

^{1}H NMR (400 MHz, $CDCl_3$) δ 1.51 (s, 9H), 6.58 (brs, 1H), 6.74 (d, 1H, J = 8.0 Hz), 7.07 (d, 1H, J = 7.6 Hz), 7.19 and 7.31 (ABq, 2H, J = 9.4 Hz), 7.25 (dd, 1H, J = 7.6, 8.0 Hz), 7.37 (brs, 1H), 7.81 (brs, 1H), 11.86 (s, 1H), 12.83 (s, 1H); ^{13}C NMR (100 MHz, $CDCl_3$) δ 28.3, 81.0, 110.2, 110.3, 114.0, 114.3, 115.8, 119.2, 127.6, 128.7, 131.5, 137.7, 138.7, 142.1, 152.5, 157.0, 158.4, 181.4, 182.1; HRMS: ESI, m/z, [M–H]$^-$, Calcd. For $C_{21}H_{19}ClN_2O_6$: 429.0859, Found: 429.0861.

5-((3-chloro-5,8-dihydroxy-1,4-dioxo-1,4-dihydronaphthalen-2-yl)amino)-2-hydroxybenzoic acid (2m): prepared by Method B (at 50 °C for 12 hours in acetonitrile), 15 % yield.

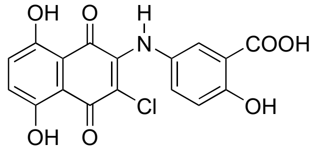

^{1}H NMR (400 MHz, DMSO-d_6) δ 6.95 (d, 1H, J = 8.8 Hz), 7.25 and 7.38 (ABq, 2H, J = 9.3 Hz), 7.32 (dd, 1H, J = 2.5, 8.8 Hz), 7.59 (d, 1H, J = 2.5 Hz), 9.57 (s, 1H), 11.00–11.40 (br, 1H), 11.67 (s, 1H), 11.91 (s, 1H), 12.88 (s, 1H); ^{13}C NMR (150 MHz, DMSO-d_6) δ 110.7, 110.9, 112.1, 116.6, 126.3, 127.2, 129.8, 130.2, 132.8, 142.0, 144.2, 155.3, 156.5, 158.8, 171.6, 181.1, 182.2; HRMS: ESI, m/z, [M–H]$^-$, Calcd. For $C_{17}H_{10}ClNO_7$: 374.0073, Found: 374.0083.

5-((3-chloro-5-nitro-1,4-dioxo-1,4-dihydronaphthalen-2-yl)amino)-2-hydroxybenzoic acid (2n): prepared by Method B (at 50 °C for 7 hours in acetonitrile), 53 % yield.

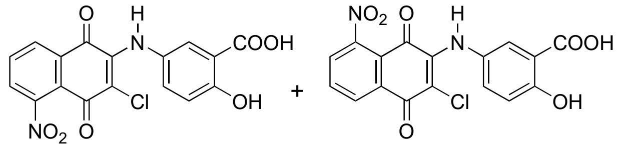

a mixture of regioisomers (1.3:1 ratio), ^{1}H NMR (400 MHz, DMSO-d_6) for major isomer: δ 6.80 (d, 1H, J = 8.6 Hz), 7.22 (dd, 1H, J = 2.7, 8.6 Hz), 7.53 (d, 1H, J = 2.7 Hz), 7.93 (t, 1H, J = 7.8 Hz), 8.07 (dd, 1H, J = 1.2, 7.8 Hz), 8.20 (dd, 1H, J = 1.2, 7.8 Hz), 9.49 (s, 1H), 11.00–13.20 (br, 2H); for minor isomer: δ 6.80 (d, 1H, J = 8.6 Hz), 7.21 (dd, 1H, J = 2.7, 8.6 Hz), 7.51 (d, 1H, J = 2.7 Hz), 8.01 (t, 1H, J = 7.8 Hz), 8.09 (dd, 1H, J = 1.2, 7.8 Hz), 8.21 (dd, 1H, J = 1.2, 7.8 Hz), 9.40 (s, 1H), 11.00–13.20 (br, 2H);
^{13}C NMR (150 MHz, DMSO-d_6) for regioisomer mixture: δ 111.4, 112.0, 114.6, 115.2, 121.3, 122.7, 126.1, 126.4, 126.6, 128.0, 128.4, 128.8, 129.1, 129.3, 131.2, 131.4, 131.6, 133.1, 134.2, 135.8, 143.9, 144.0, 147.8, 148.0, 159.2, 159.4, 170.4, 171.8, 173.3, 174.8, 177.4, 178.5; HRMS: ESI, m/z, [M+H]$^+$, Calcd. For $C_{17}H_9ClN_2O_7$: 389.0171, Found: 389.0167.

Synthesis of 2-aminonaphthoquinone 2o

Naphthoquinone **2n** (122 mg, 0.31 mmol) was dissolved in ethanol (4 mL), then zinc powder (830 mg, 4 equiv.) and 6N-HCl (4 mL) were added. The reaction was stirred at room temperature for 20 hours and quenched by the addition of $NaHCO_3$ (1.0 g). The mixture was dried over Na_2SO_4 and filterd through a cotton pad. The obtained filtrate was concentrated and purified by column chromatography to give 2-aminonaphthoquinone **2o** as purple solid (65 mg, 44 %).

5-((5-amino-3-chloro-1,4-dioxo-1,4-dihydronaphthalen-2-yl)amino)-2-hydroxybenzoic acid (2o)

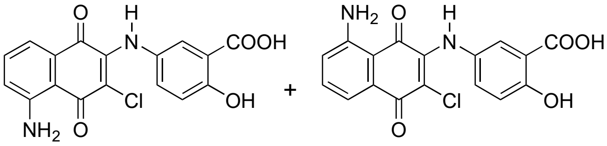

a mixture of regioisomers (1.3:1 ratio), ^{1}H NMR (400 MHz, DMSO-d_6) for major isomer: δ 6.90 (d, 1H, J = 8.8 Hz), 7.15 (d, 1H, J = 8.4 Hz), 7.22–7.35 (m, 2H), 7.40 (d, 1H, J = 7.6 Hz), 7.48 (d, 1H, J = 2.7 Hz), 7.50–8.04 (br, 2H), 8.87 (s, 1H), 10.80–11.30 (br, 2H); for minor isomer: δ 6.92 (d, 1H, J = 8.8 Hz), 7.10 (d, 1H, J = 8.4 Hz), 7.22–7.35 (m, 2H), 7.42 (d, 1H, J = 7.6 Hz),, 7.51 (d, 1H, J = 2.7 Hz), 7.50–8.04 (br, 2H), 9.14 (s, 1H), 10.80–11.30 (br, 2H); ^{13}C NMR (150 MHz, DMSO-d_6) for regioisomer mixture: δ 108.6, 109.6, 110.8, 112.0, 114.8, 115.8, 116.3, 116.5, 122.6, 124.6, 125.0, 125.9, 130.3, 130.8, 131.2, 132.0, 132.5, 132.7, 133.3, 135.2, 142.0, 143.9, 150.6, 152.0, 158.0, 158.4, 170.4, 171.7, 176.6, 179.3, 179.7, 180.3; HRMS: ESI, m/z, [M–H]$^-$, Calcd. For $C_{17}H_{11}ClN_2O_5$: 357.0284, Found: 357.0273.

Synthesis of naphtho[2,3-*d*]isoxazole-4,9-diones 3

Naphthoquinone **1a** or **1b** (0.1 mmol) and an oxime (0.2mmol) were dissolved in dichloroethane (3 mL). The solution was added sodium hypochlorite (5 % solution in water, 2 mL), and stirred for 2 hours at room temperature. The mixture was concentrated and the resulting residue was purified by column chromatography to give naphtho[2,3-*d*]isoxazole-4,9-diones 3. The product structure and spectral data were listed below.

5,8-dihydroxy-3-phenylnaphtho[2,3-*d*]isoxazole-4,9-dione (**3a**): 51 % yield

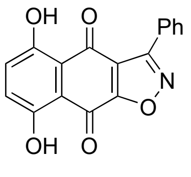

^{1}H and ^{13}C NMR data were identical for literature.[2]

HRMS: ESI, m/z, [M–H]$^-$, Calcd. For $C_{17}H_9NO_5$: 306.0408, Found: 306.0405.

5,8-dihydroxy-3-methylnaphtho[2,3-*d*]isoxazole-4,9-dione (**3b**): 53 % yield

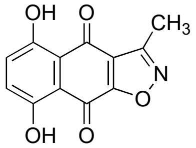

^{1}H NMR (400 MHz, $CDCl_3$) δ 2.69 (s, 3H), 7.31 and 7.36 (ABq, 2H, J = 9.5 Hz), 12.38 (s, 1H), 12.69 (s, 1H); ^{13}C NMR (150 MHz, $CDCl_3$) δ 10.9, 112.2, 112.8, 121.2, 130.2, 132.1, 158.4, 158.8, 160.1, 165.1, 175.8, 183.5; HRMS: ESI, m/z, [M–H]⁻, Calcd. For $C_{12}H_7NO_5$: 244.0251, Found: 244.0254.

8-hydroxy-3-phenylnaphtho[2,3-*d*]isoxazole-4,9-dione (**3c**): 55 % yield

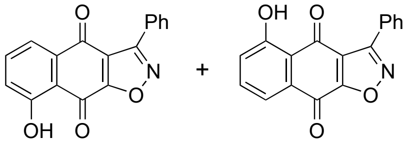

(major)

a mixture of regioisomers (3:1 ratio): major isomer was isolated by recrystallization from the mixed solvent of hexane and ethyl acetate (5:1, v/v). The structural determination was conducted by the similar manner as that for reported procedure. [2]

^{1}H NMR (400 MHz, $CDCl_3$) for major isomer: δ 7.36 (dd, 1H, J = 1.2, 8.4 Hz), 7.50–7.60 (m, 3H), 7.72 (dd, 1H, J = 7.4, 8.4 Hz), 7.80 (dd, 1H, J = 1.2, 7.4 Hz), 8.12–8.19 (m, 2H), 11.72 (s, 1H); for minor isomer: δ 7.38 (d, 1H, J = 8.3 Hz), 7.50–7.60 (m, 3H), 7.67 (dd, 1H, J = 7.4, 8.3 Hz), 7.82 (dd, 1H, J = 1.2, 7.4 Hz), 8.07–8.12 (m, 2H), 12.23 (s, 1H); ^{13}C NMR (150 MHz, $CDCl_3$) for major isomer: δ 114.8, 120.1, 120.9, 125.2, 125.9, 128.7, 129.3, 131.4, 133.9, 138.0, 161.1, 163.2, 166.0, 177.7, 177.9; HRMS: ESI, m/z, [M–H]⁻, Calcd. For $C_{17}H_9NO_4$: 290.0459, Found: 290.0464.

1. Ravichandiran P, Subramaniyan SA, Kim S-Y, Kim J-S, Park B-H, Shim KS, Yoo DJ, Synthesis and Anticancer Evaluation of 1,4-Naphthoquinone Derivatives Containing a Phenylaminosulfanyl Moiety, *Chem. Med. Chem*, 2019; 14: 532–544.

2. Fariña F, Martin MV, Muñoz M, Paredes MC, Rodorigues R, *Heterocycles*, 1,3-Dipolar cycloaddition of nitrile oxides to 1,4-naphthoquinone derivatives, 1995; 40: 413–424.

NMR charts of the synthesized compounds were listed bellow.

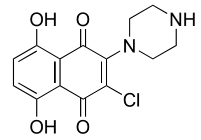
OH
O
NH
N
Cl
OH
O

2a

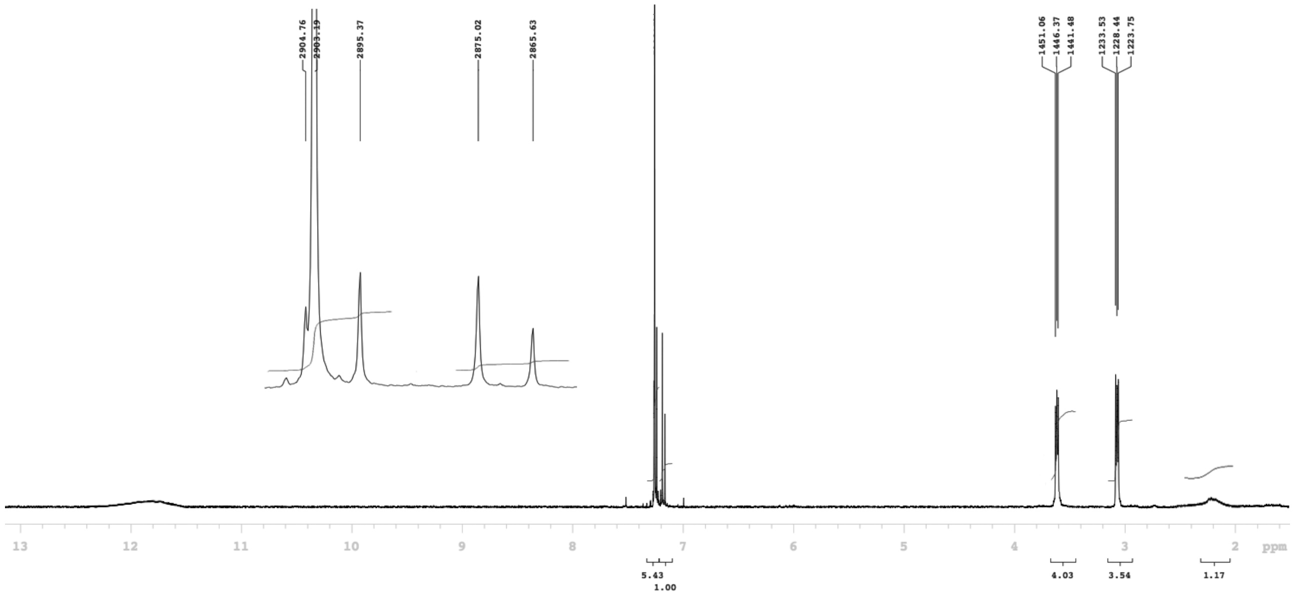
2904.76
2903.19
2895.37
2875.02
2865.63
1451.06
1446.37
1441.48
1233.53
1228.44
1223.75
13 12 11 10 9 8 7 6 5 4 3 2 ppm
5.43
1.00
4.03
3.54
1.17

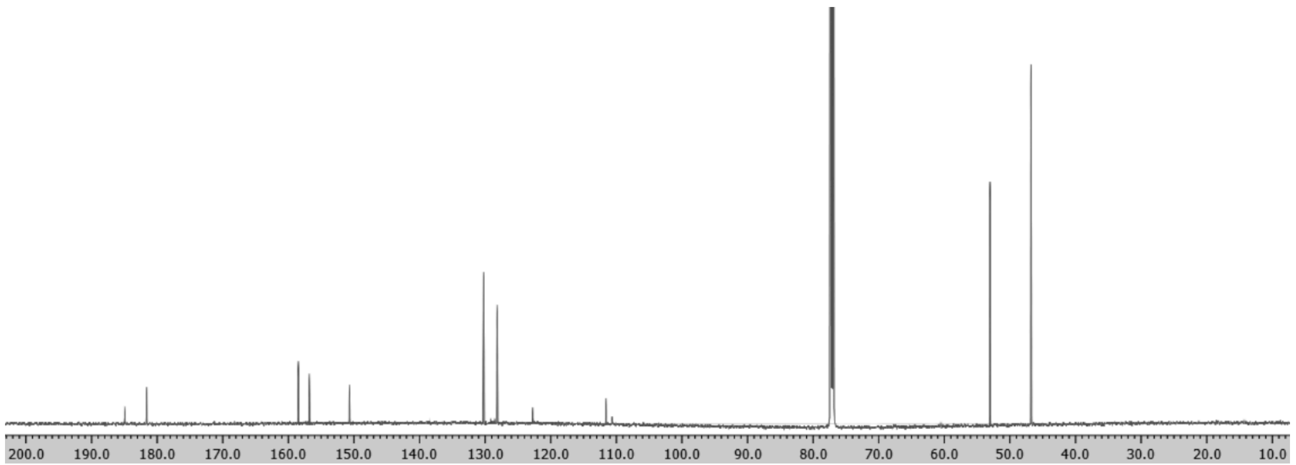
200.0 190.0 180.0 170.0 160.0 150.0 140.0 130.0 120.0 110.0 100.0 90.0 80.0 70.0 60.0 50.0 40.0 30.0 20.0 10.0

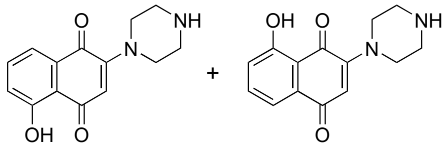

O
N
NH
OH
O
+
OH
O
N
NH
O

2b

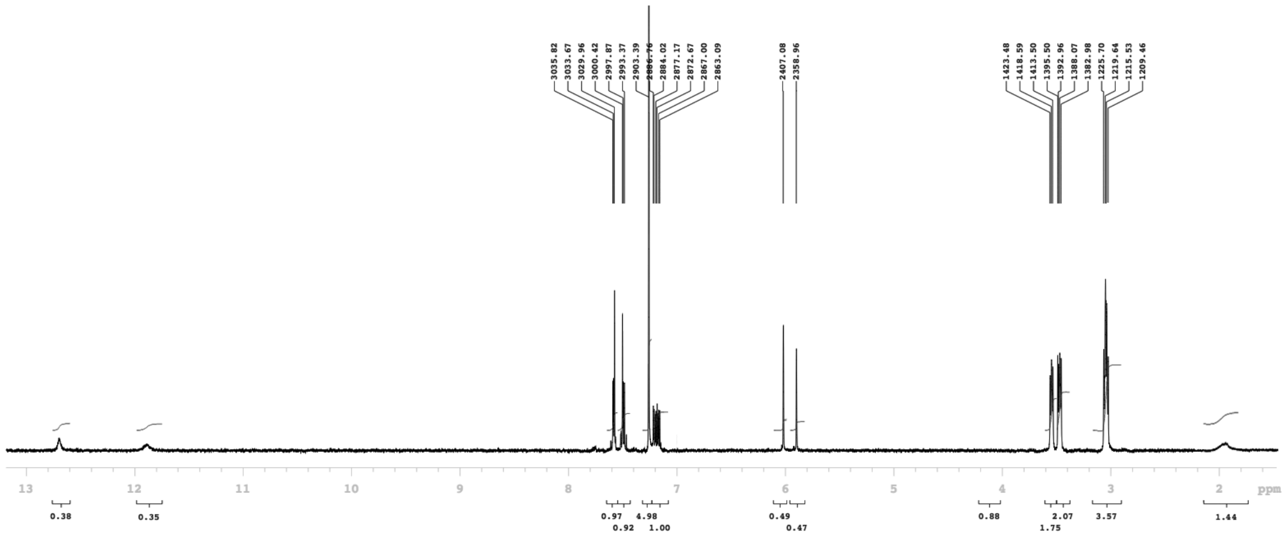

3035.82
3033.67
3029.96
3000.42
2997.87
2993.37
2903.39
2884.02
2877.17
2872.67
2867.00
2863.09
2407.08
2358.96
1423.48
1418.59
1413.50
1395.50
1392.96
1388.07
1382.98
1225.70
1219.64
1215.53
1209.46
13 12 11 10 9 8 7 6 5 4 3 2 ppm
0.38
0.35
0.97
0.92
4.98
1.00
0.49
0.47
0.88
1.75
2.07
3.57
1.44

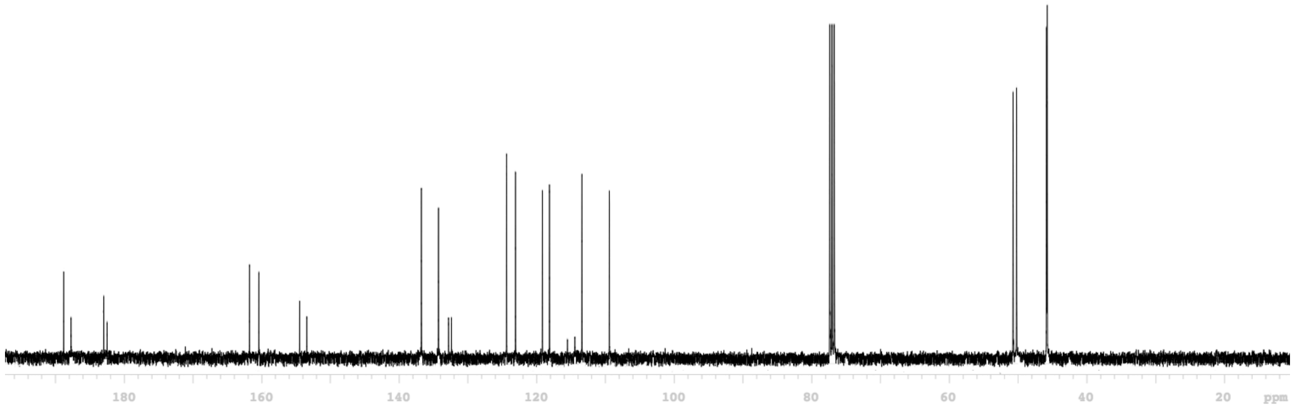

180 160 140 120 100 80 60 40 20 ppm

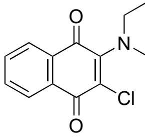
O
NH
N
Cl
O

2c

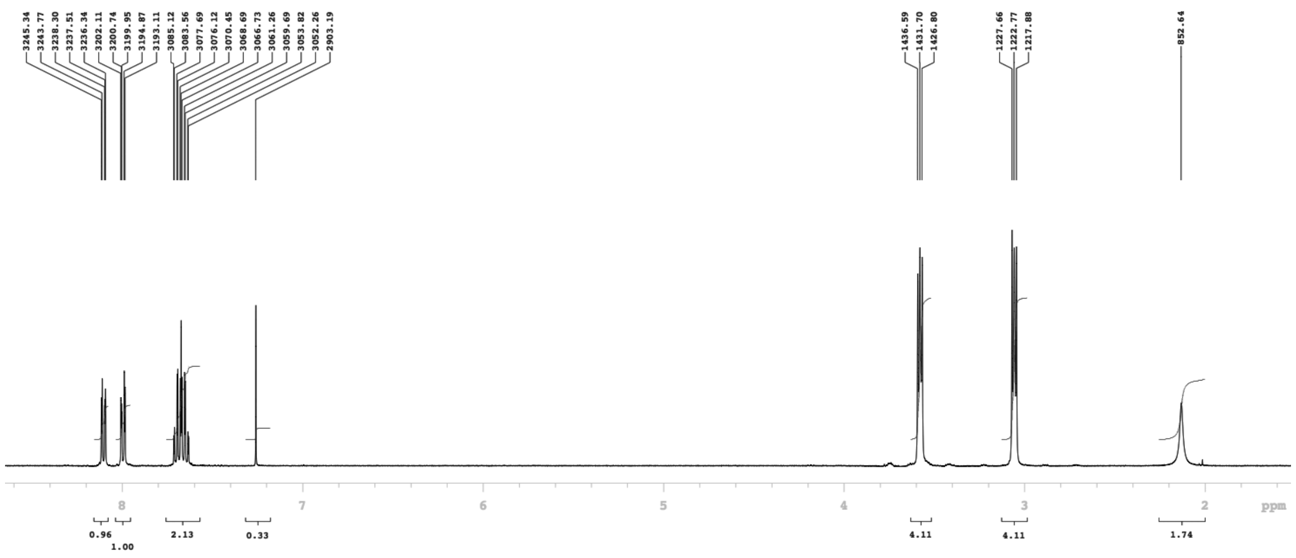
3245.34
3243.77
3238.30
3237.51
3236.34
3202.11
3200.74
3199.95
3194.87
3193.11
3085.12
3083.56
3077.69
3076.12
3070.45
3068.69
3066.73
3061.26
3059.69
3053.82
3052.26
2903.19
1436.59
1431.70
1426.80
1227.66
1222.77
1217.88
852.64
8
7
6
5
4
3
2
ppm
0.96
1.00
2.13
0.33
4.11
4.11
1.74

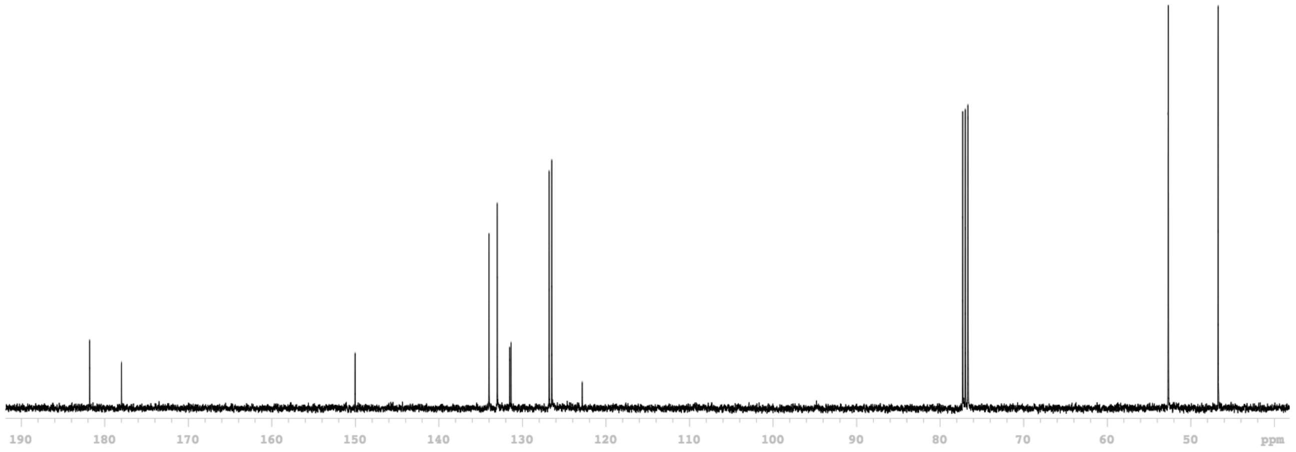
190
180
170
160
150
140
130
120
110
100
90
80
70
60
50
ppm

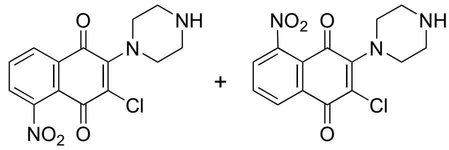

O
NH
N
Cl
NO2
+

2d

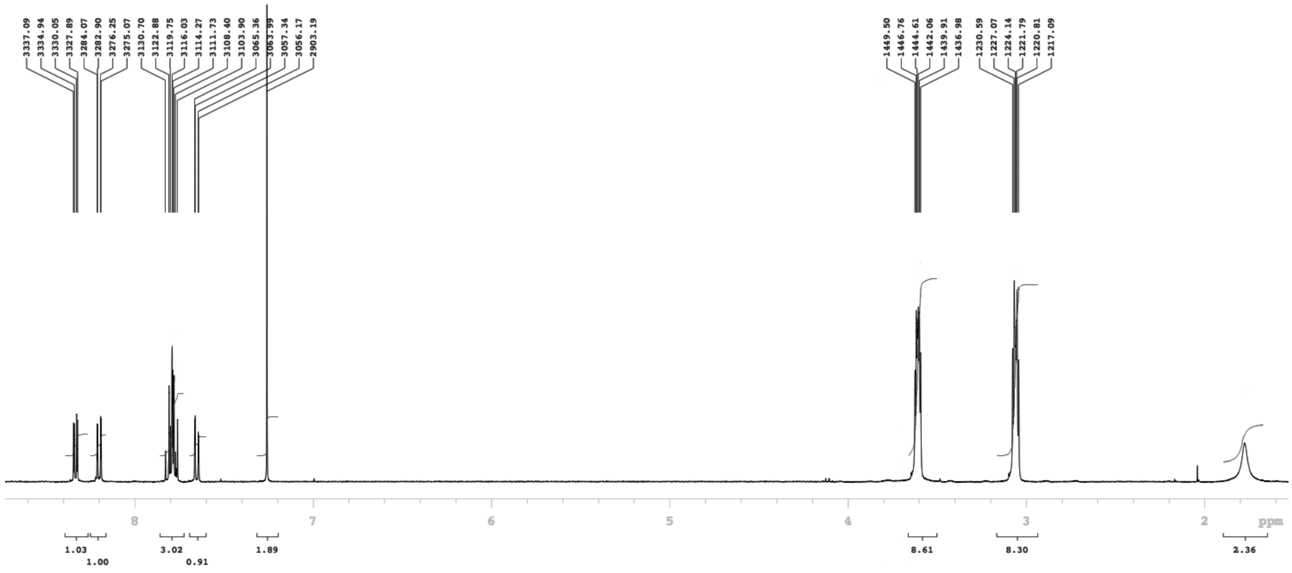

3337.09
3334.94
3330.05
3327.89
3284.07
3282.90
3276.25
3275.07
3130.70
3122.88
3119.75
3116.03
3114.27
3111.73
3108.40
3103.90
3065.36
3063.99
3057.34
3056.17
2903.19
1449.50
1446.76
1444.61
1442.06
1439.91
1436.98
1230.59
1227.07
1224.14
1221.79
1220.81
1217.09
8
7
6
5
4
3
2
ppm
1.03
1.00
3.02
0.91
1.89
8.61
8.30
2.36

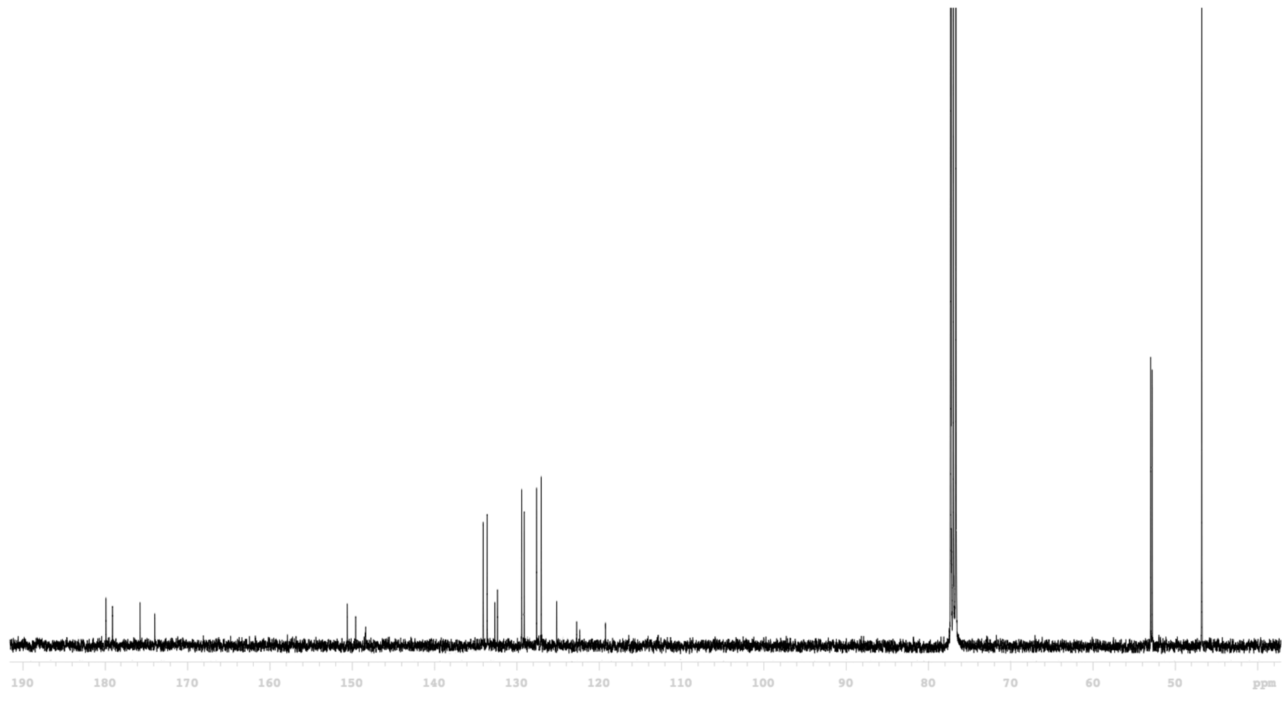

190
180
170
160
150
140
130
120
110
100
90
80
70
60
50
ppm

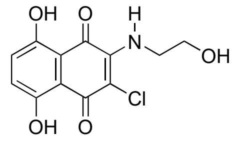
OH
O
H
N
OH
Cl
OH
O

2e

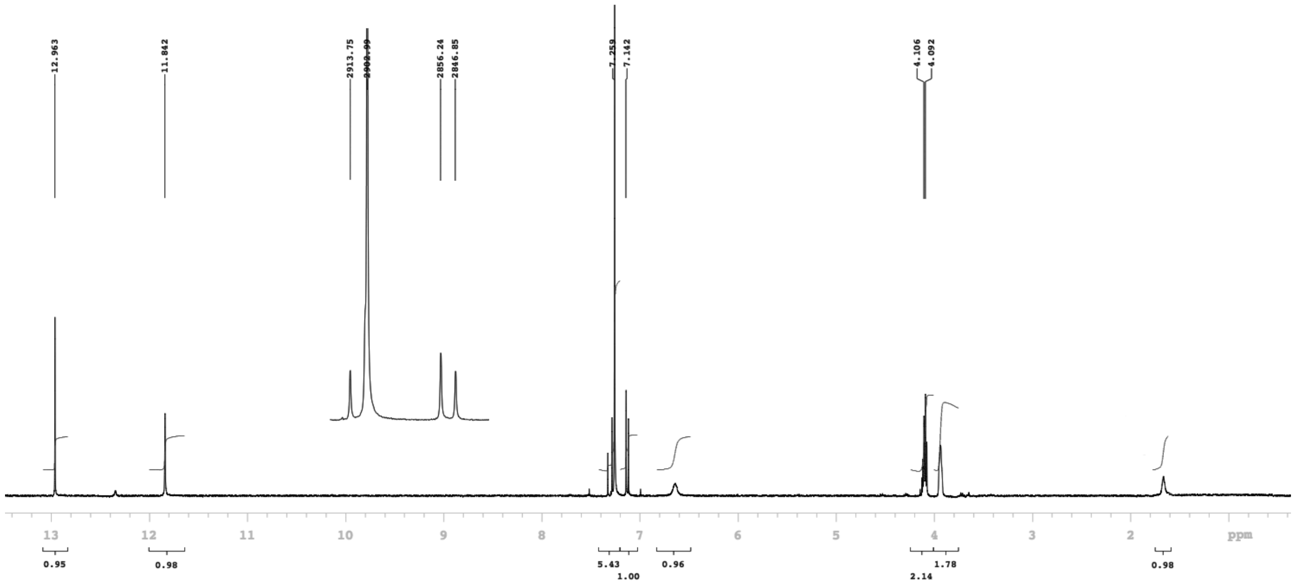
12.963
11.842
2913.75
2902.99
2856.24
2846.85
7.259
7.142
4.106
4.092
13
12
11
10
9
8
7
6
5
4
3
2
ppm
0.95
0.98
5.43
1.00
0.96
2.14
1.78
0.98

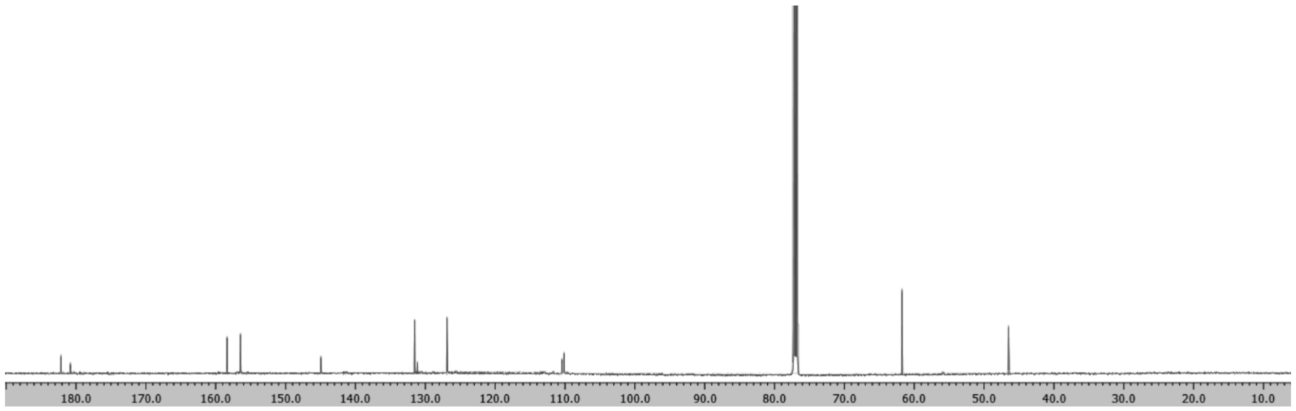
180.0
170.0
160.0
150.0
140.0
130.0
120.0
110.0
100.0
90.0
80.0
70.0
60.0
50.0
40.0
30.0
20.0
10.0

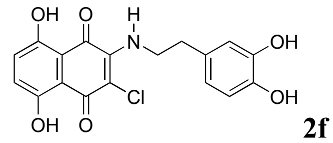

2f

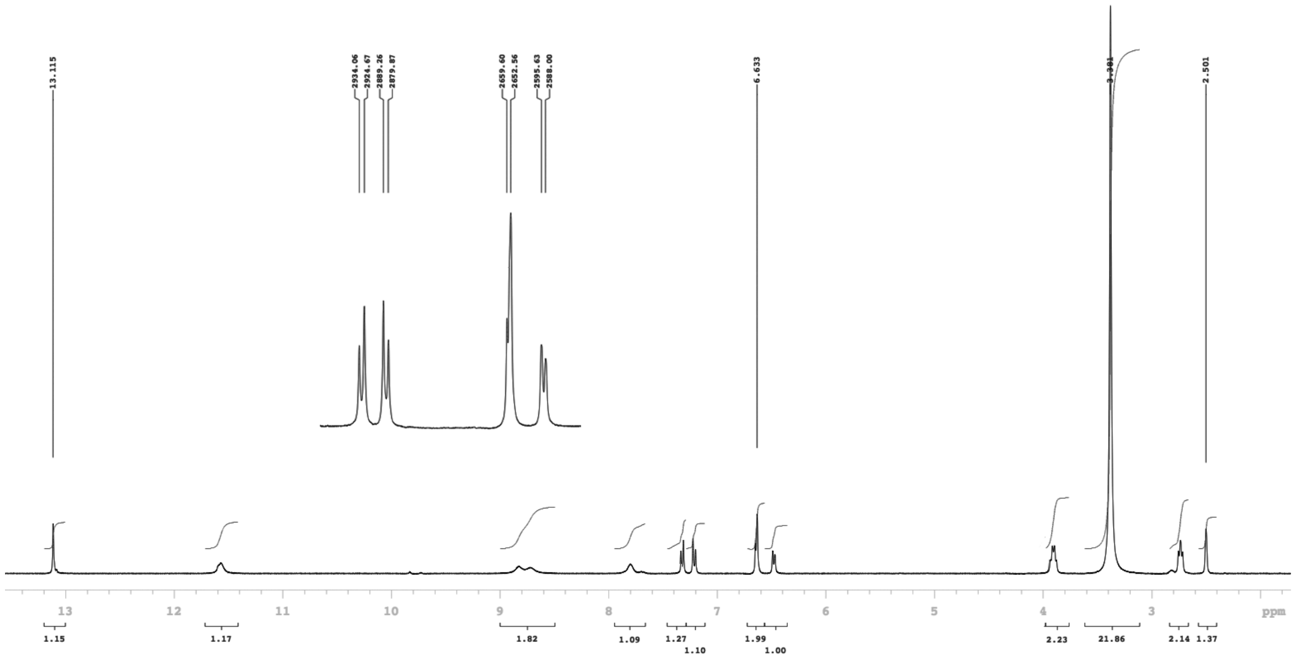

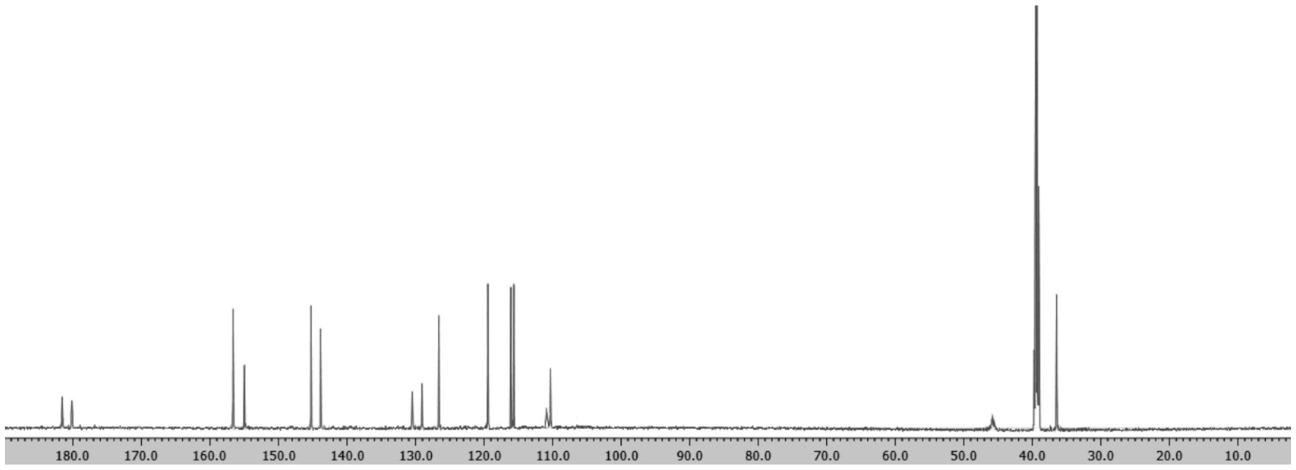

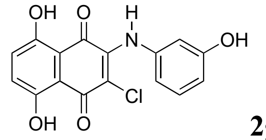
OH
O
H
N
OH
Cl
OH
O

2g

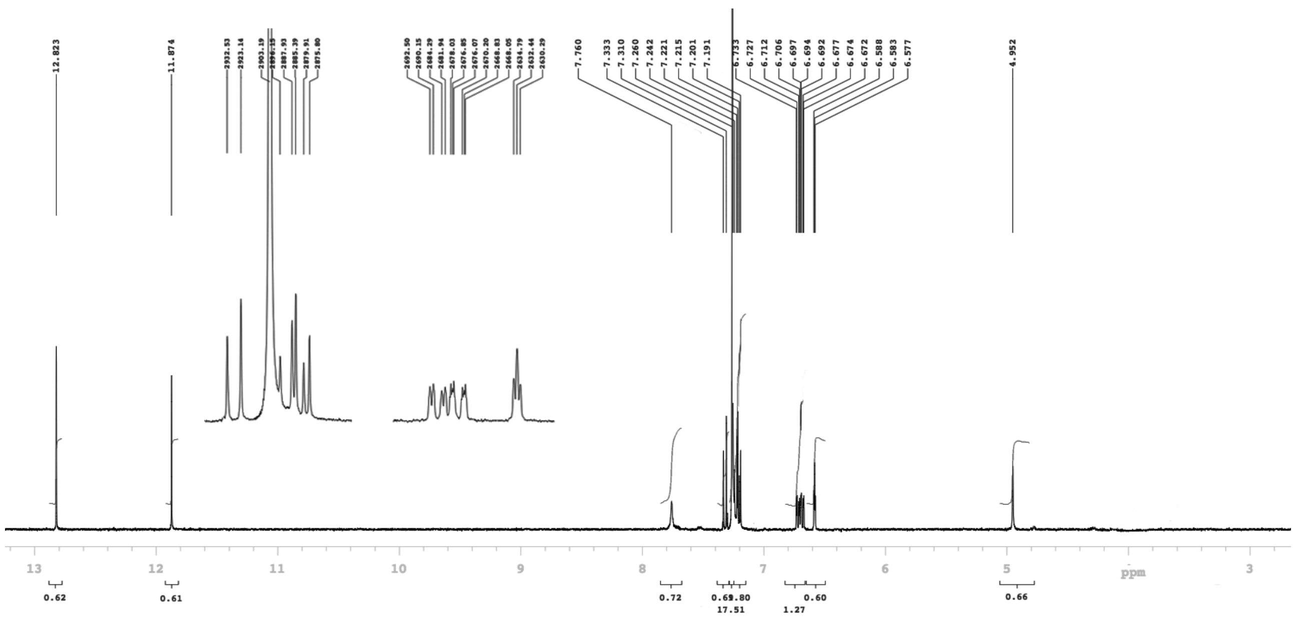
12.823
11.874
7.760
7.333
7.310
7.260
7.242
7.221
7.215
7.201
7.191
6.733
6.727
6.712
6.706
6.697
6.694
6.692
6.677
6.674
6.672
6.588
6.583
6.577
4.952
13
12
11
10
9
8
7
6
5
ppm
3
0.62
0.61
0.72
17.51
0.60
1.27
0.66

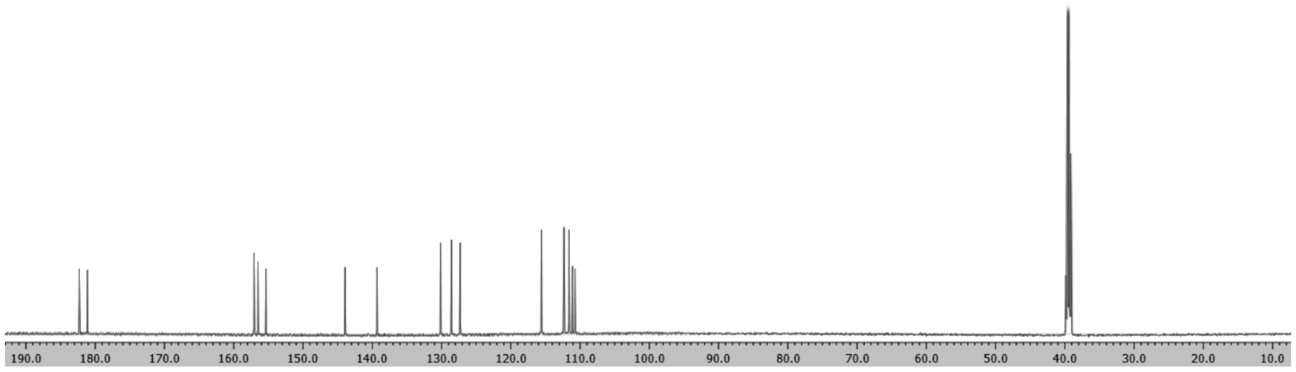
190.0
180.0
170.0
160.0
150.0
140.0
130.0
120.0
110.0
100.0
90.0
80.0
70.0
60.0
50.0
40.0
30.0
20.0
10.0

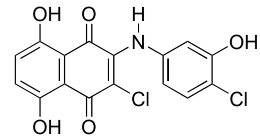
OH
O
H
N
OH
Cl
Cl
OH
O

2h

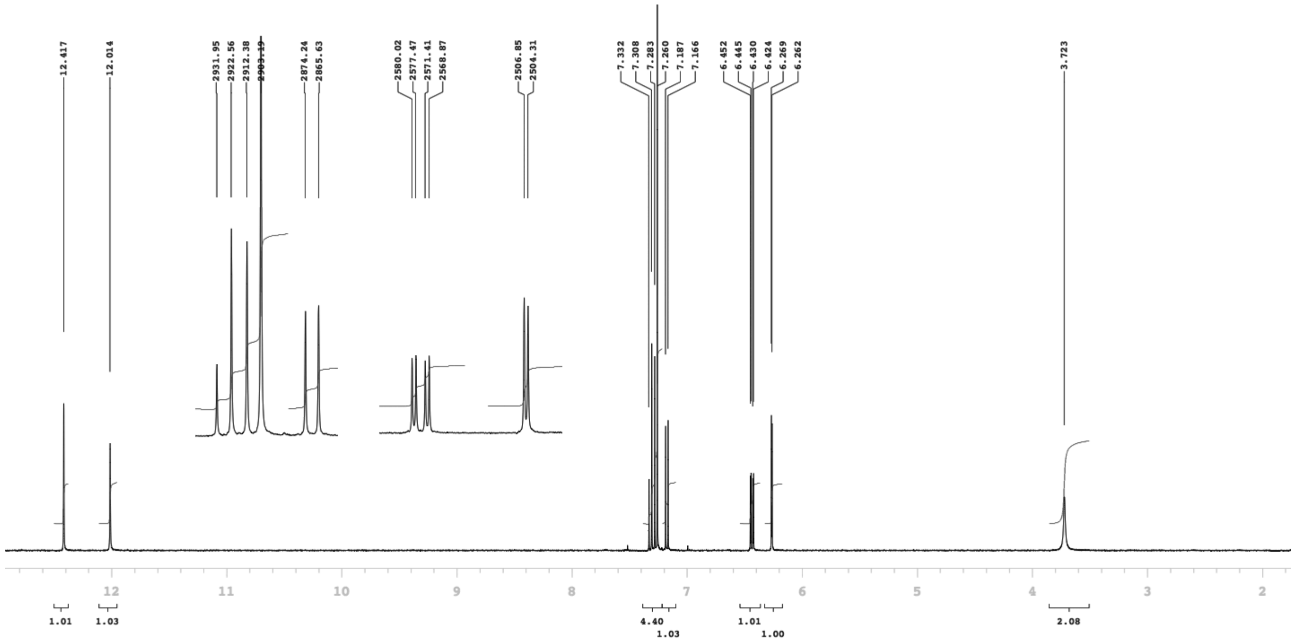
12.417
12.014
2931.95
2922.56
2912.38
2903.19
2874.24
2865.63
2580.02
2577.47
2571.41
2568.87
2506.85
2504.31
7.332
7.308
7.283
7.260
7.187
7.166
6.452
6.445
6.430
6.424
6.269
6.262
3.723
12
11
10
9
8
7
6
5
4
3
2
1.01
1.03
4.40
1.03
1.01
1.00
2.08

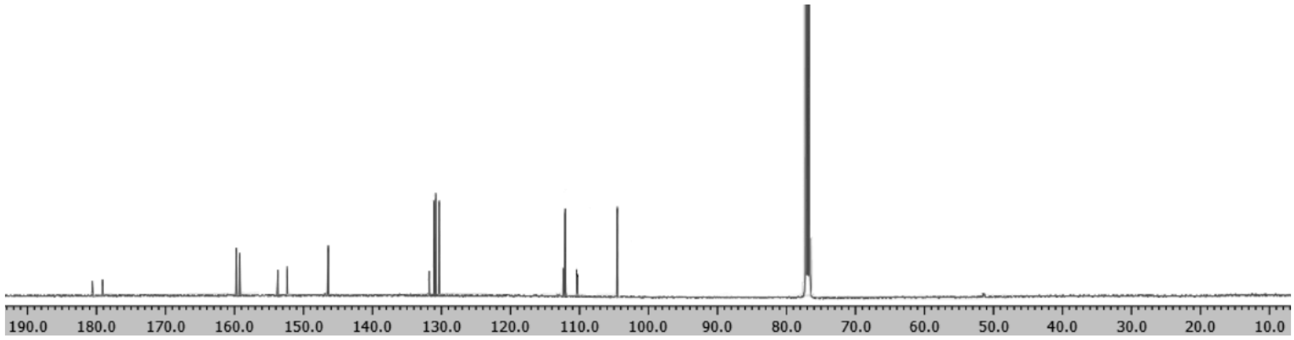
190.0
180.0
170.0
160.0
150.0
140.0
130.0
120.0
110.0
100.0
90.0
80.0
70.0
60.0
50.0
40.0
30.0
20.0
10.0

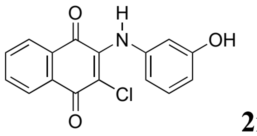
H
N
OH
O
O
Cl

2i

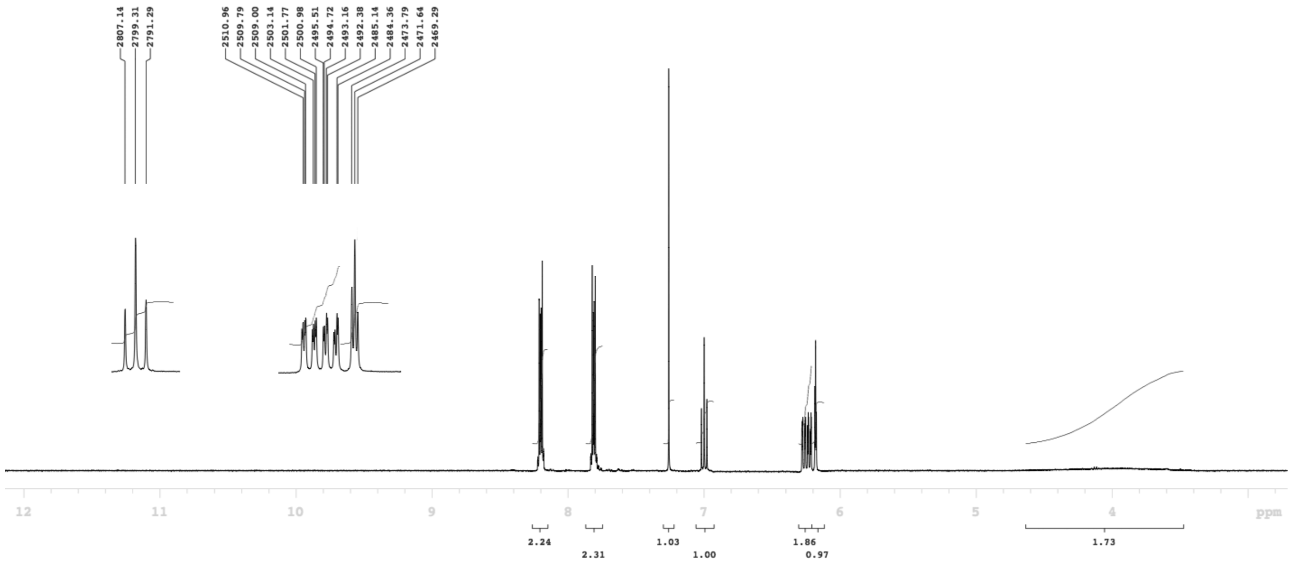
2807.14
2799.31
2791.29
2510.96
2509.79
2509.00
2503.14
2501.77
2500.98
2495.51
2494.72
2493.16
2492.38
2485.14
2484.36
2473.79
2471.64
2469.29
12
11
10
9
8
7
6
5
4
ppm
2.24
2.31
1.03
1.00
1.86
0.97
1.73

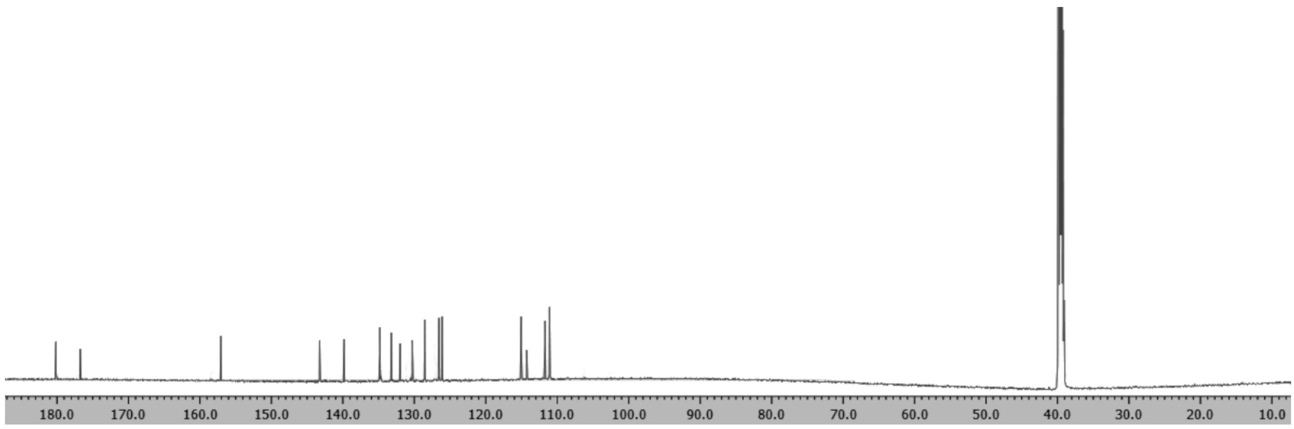
180.0
170.0
160.0
150.0
140.0
130.0
120.0
110.0
100.0
90.0
80.0
70.0
60.0
50.0
40.0
30.0
20.0
10.0

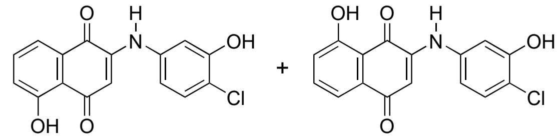
O
H
N
OH
Cl
OH
O
+
OH
O
H
N
OH
Cl
O

2j

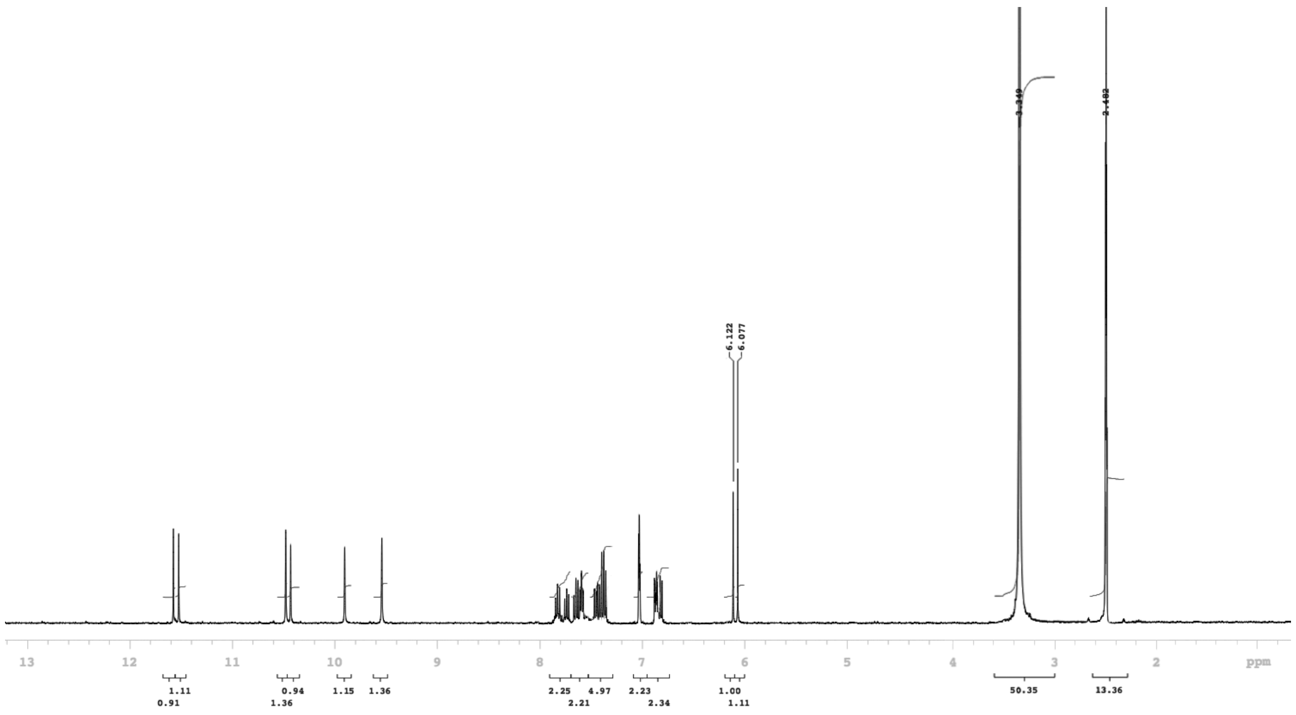
6.122
6.077
3.349
2.483
13
12
11
10
9
8
7
6
5
4
3
2
ppm
1.11
0.91
0.94
1.36
1.15
1.36
2.25
2.21
4.97
2.23
2.34
1.00
1.11
50.35
13.36

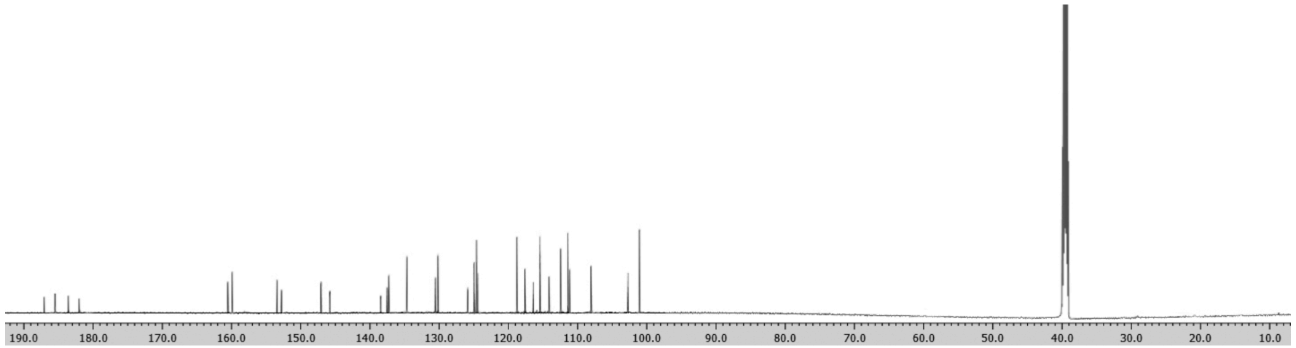
190.0
180.0
170.0
160.0
150.0
140.0
130.0
120.0
110.0
100.0
90.0
80.0
70.0
60.0
50.0
40.0
30.0
20.0
10.0

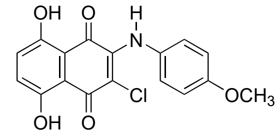
OH
O
H
N
Cl
OCH3
OH
O

2k

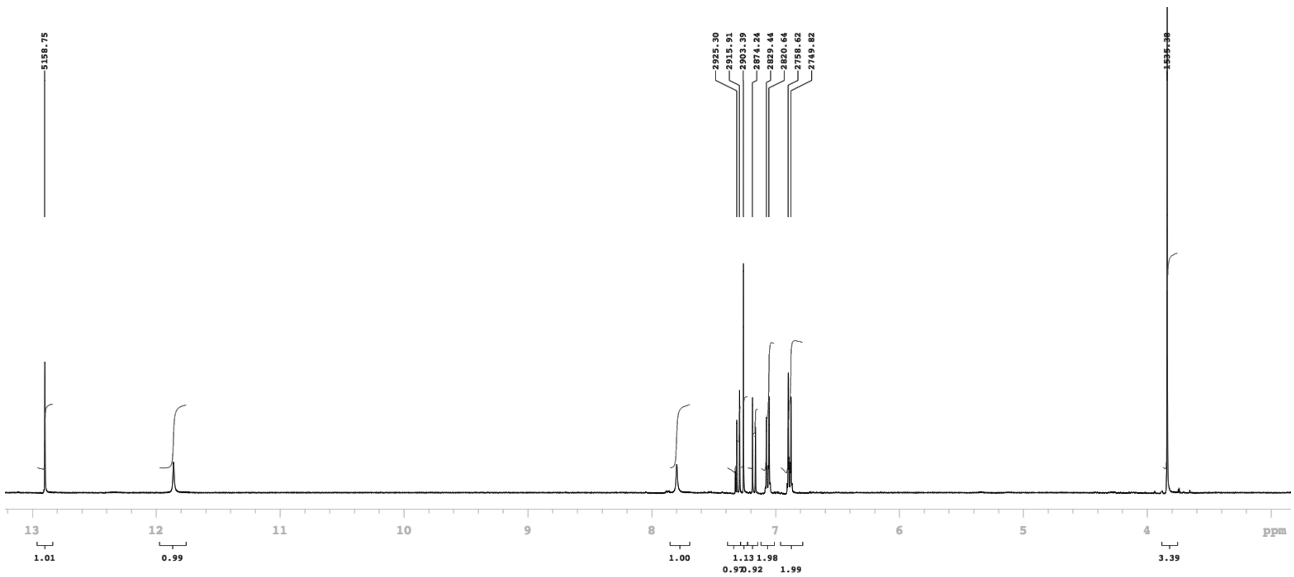
5158.75
2925.30
2915.91
2903.39
2874.24
2829.44
2820.64
2758.62
2749.82
1535.38
ppm
1.01
0.99
1.00
1.13
1.98
0.97
0.92
1.99
3.39

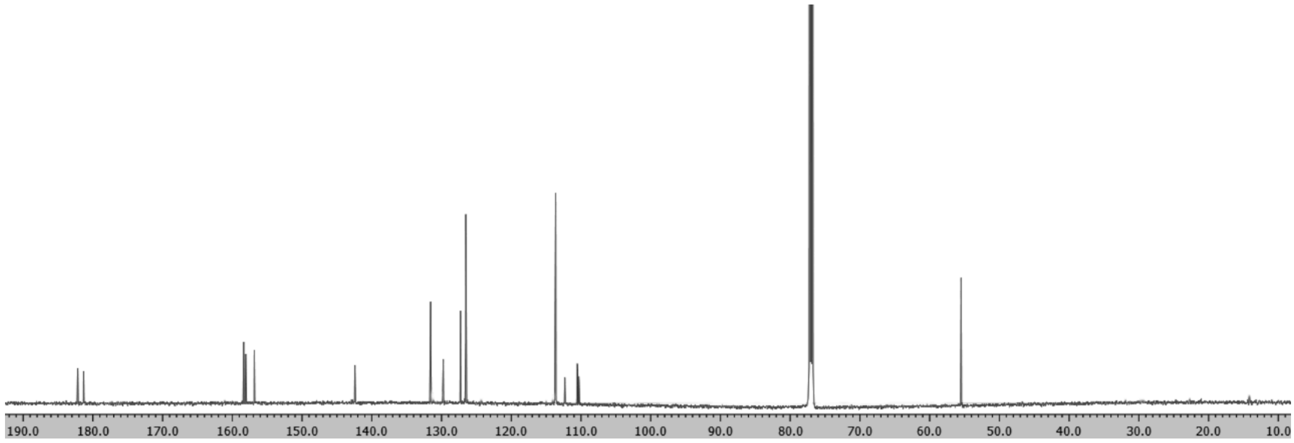
190.0
180.0
170.0
160.0
150.0
140.0
130.0
120.0
110.0
100.0
90.0
80.0
70.0
60.0
50.0
40.0
30.0
20.0
10.0

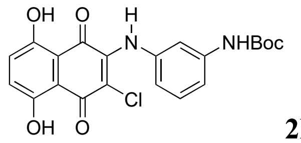
OH
O
H
N
NHBoc
Cl
OH
O

2l

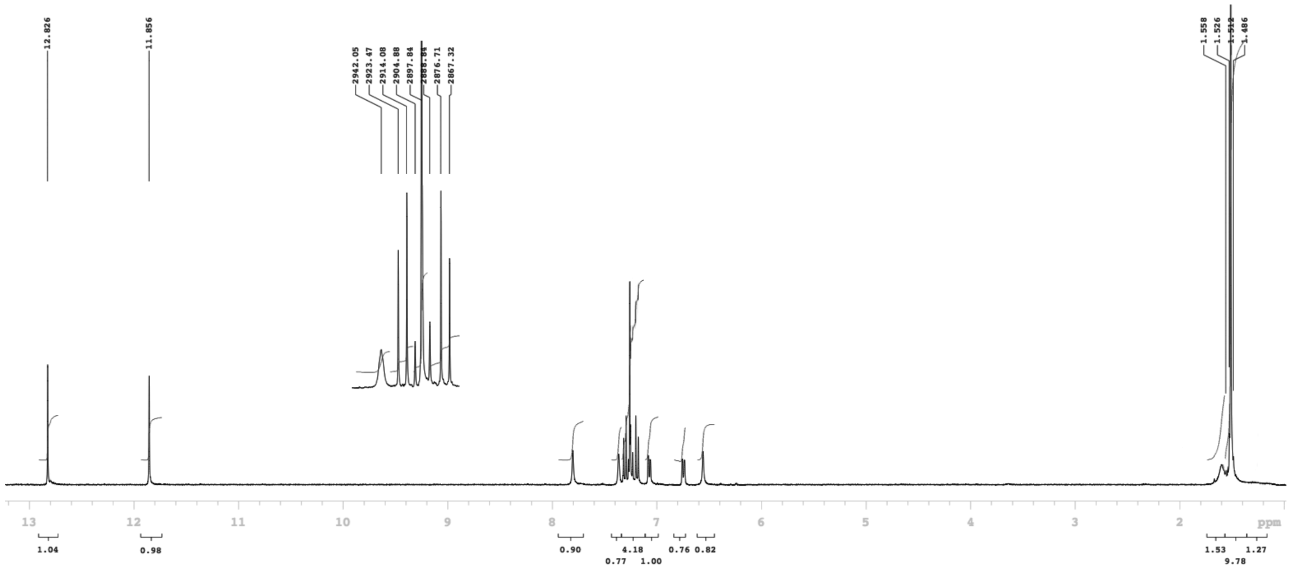
12.826
11.856
2942.05
2923.47
2914.08
2904.88
2897.84
2886.94
2876.71
2867.32
1.558
1.526
1.512
1.486
13
12
11
10
9
8
7
6
5
4
3
2
ppm
1.04
0.98
0.90
0.77
4.18
1.00
0.76
0.82
1.53
1.27
9.78

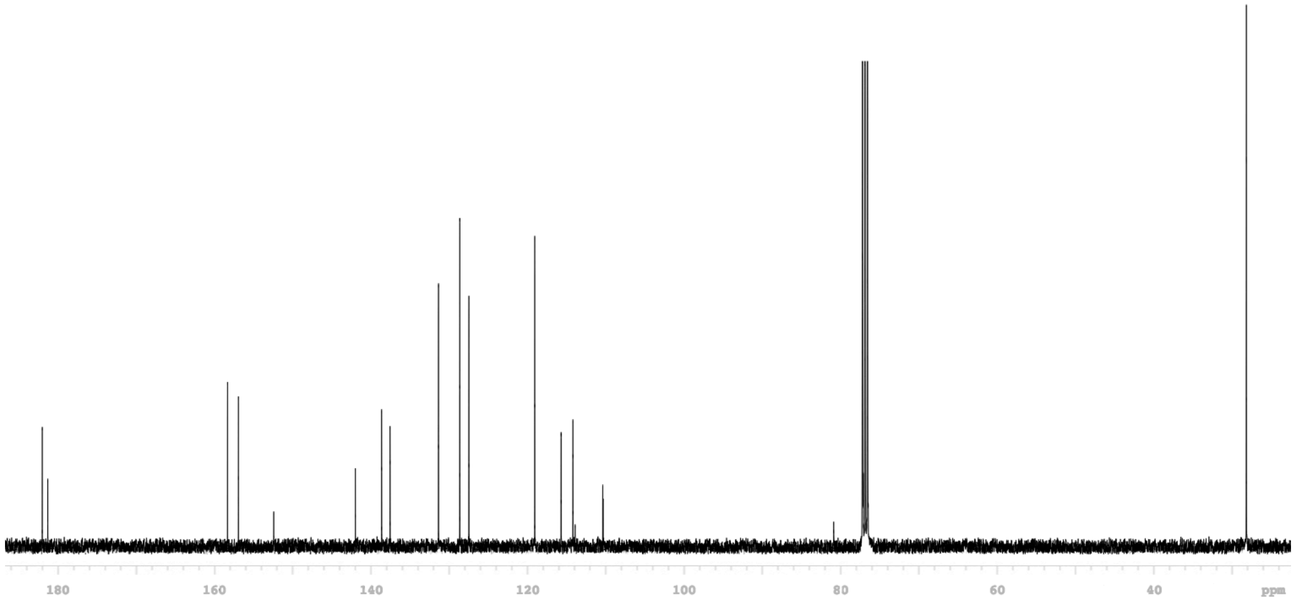
180
160
140
120
100
80
60
40
ppm

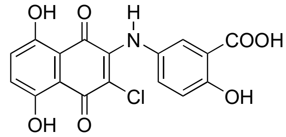
OH
O
H
N
COOH
Cl
OH
OH
O

2m

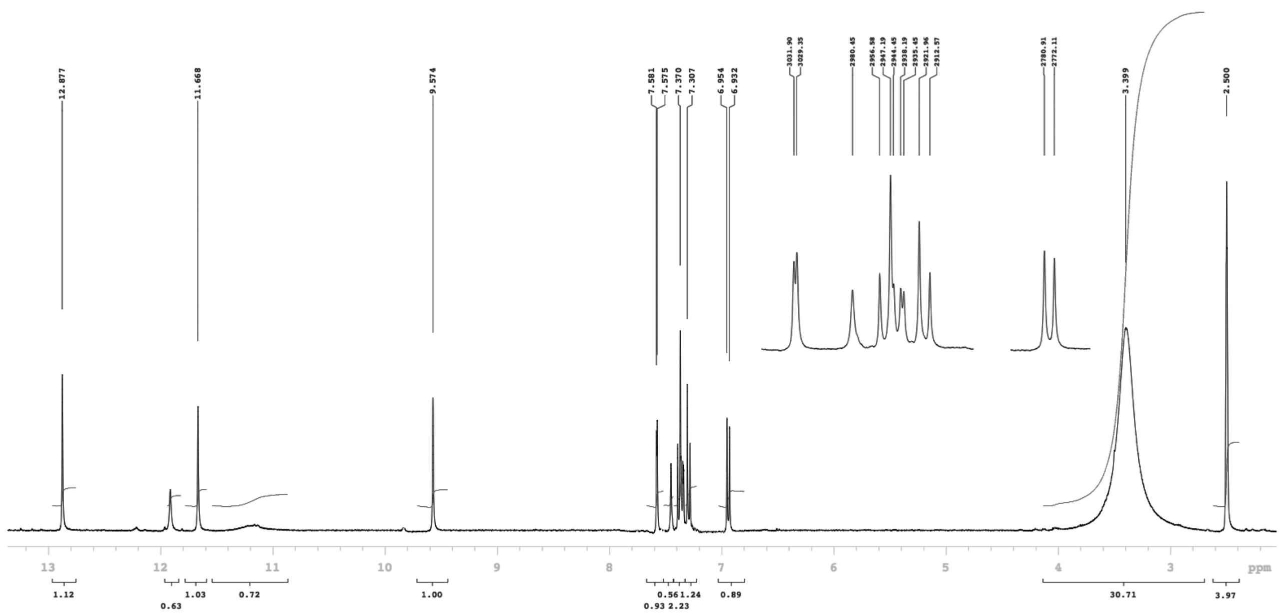
12.877
11.668
9.574
7.581
7.575
7.370
7.307
6.954
6.932
3031.90
3029.35
2980.45
2956.58
2947.19
2944.45
2938.19
2935.45
2921.96
2912.57
2780.91
2772.11
3.399
2.500
13
12
11
10
9
8
7
6
5
4
3
ppm
1.12
0.63
1.03
0.72
1.00
0.56
1.24
0.93
2.23
0.89
30.71
3.97

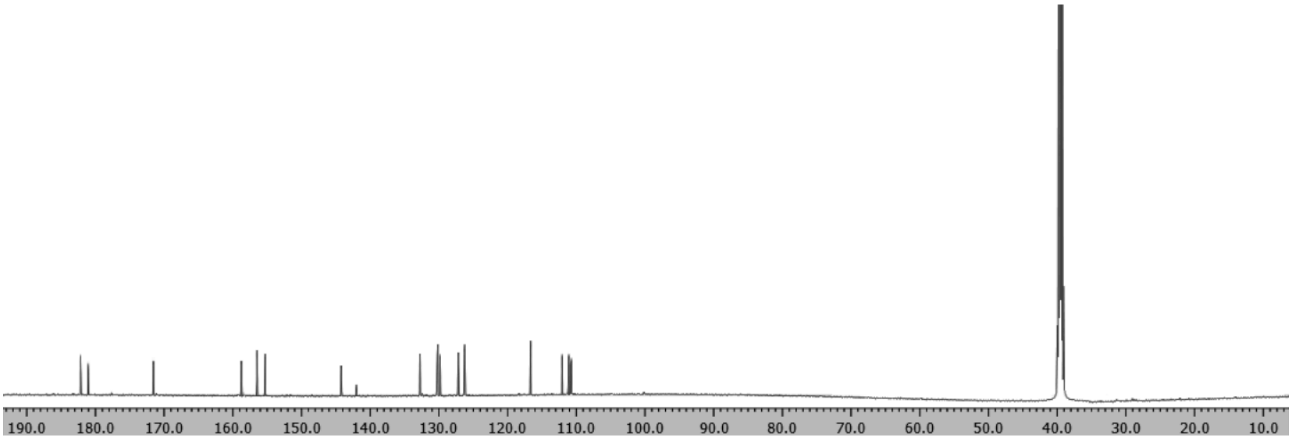
190.0
180.0
170.0
160.0
150.0
140.0
130.0
120.0
110.0
100.0
90.0
80.0
70.0
60.0
50.0
40.0
30.0
20.0
10.0

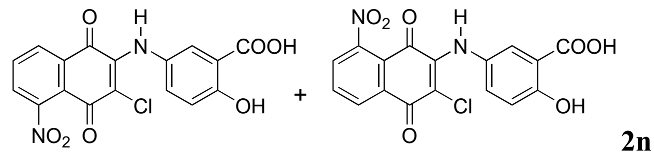

2n

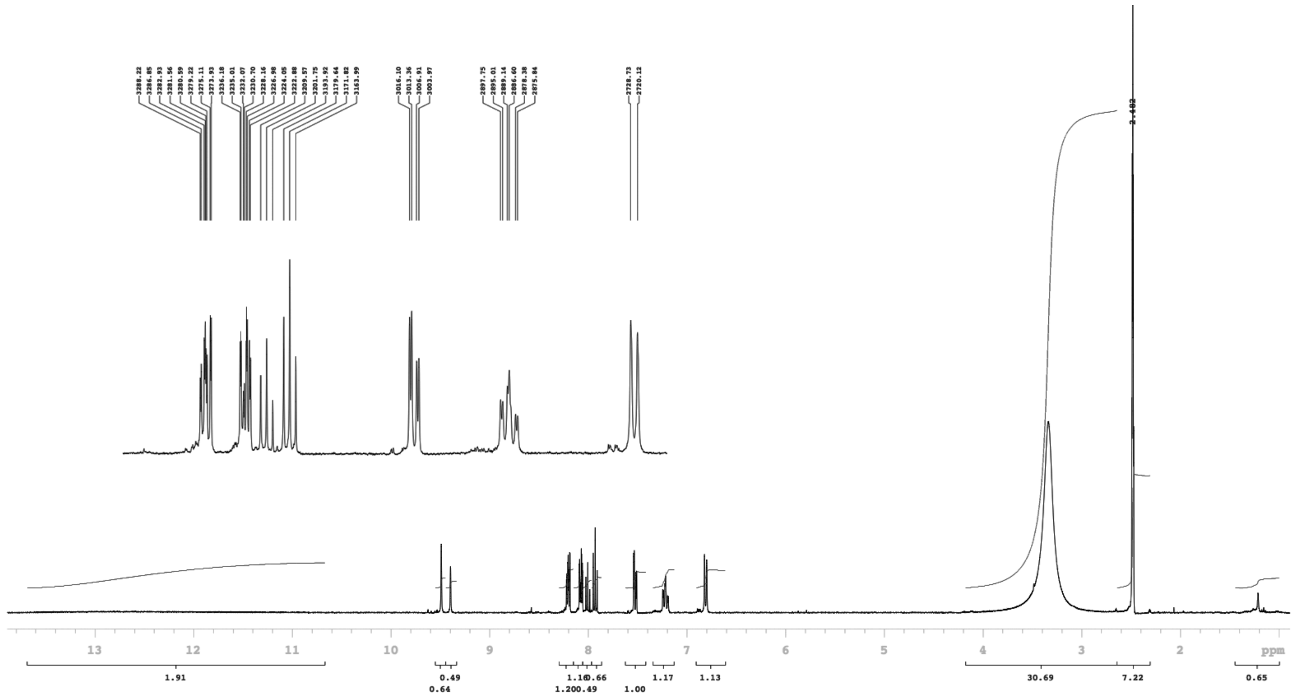

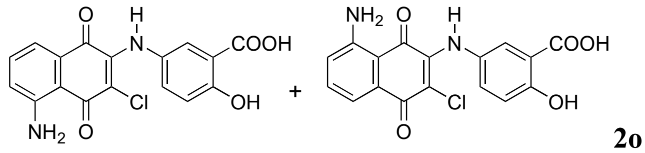

2o

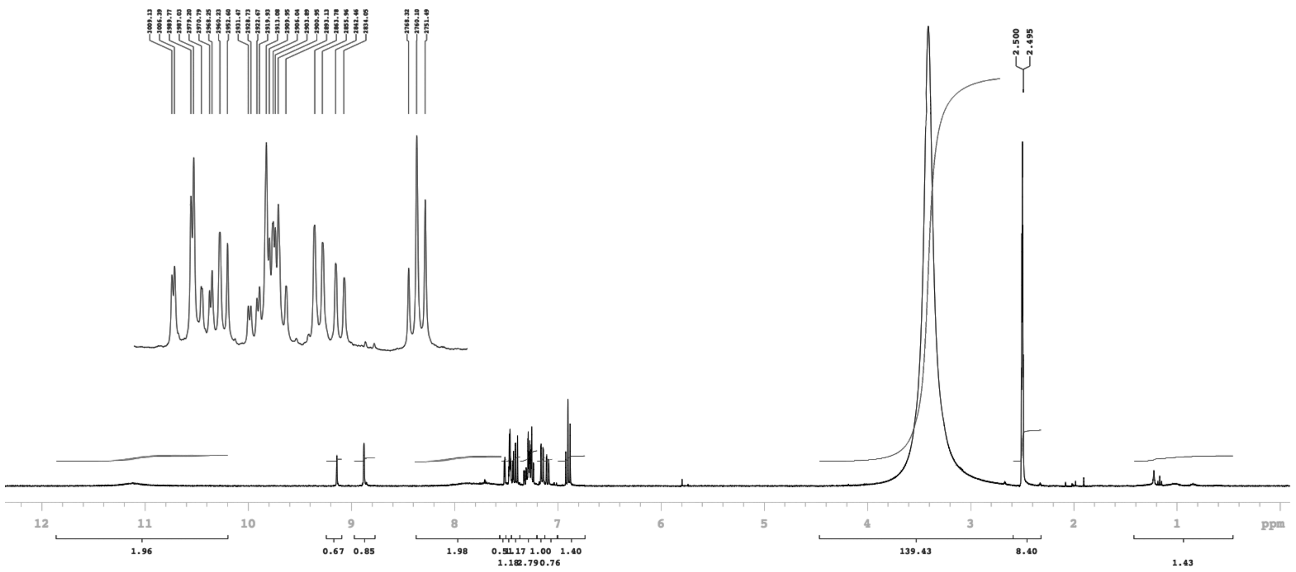

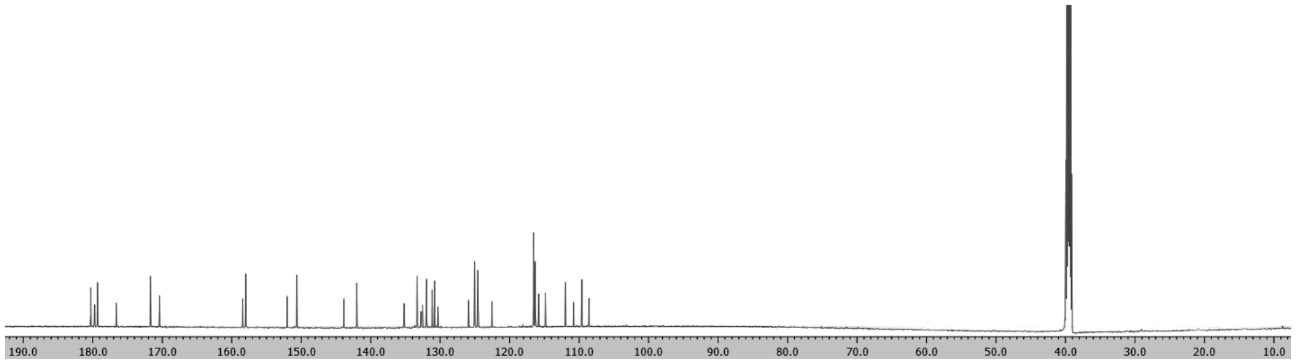

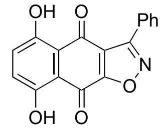
OH
O
Ph
N
O
OH
O

3a

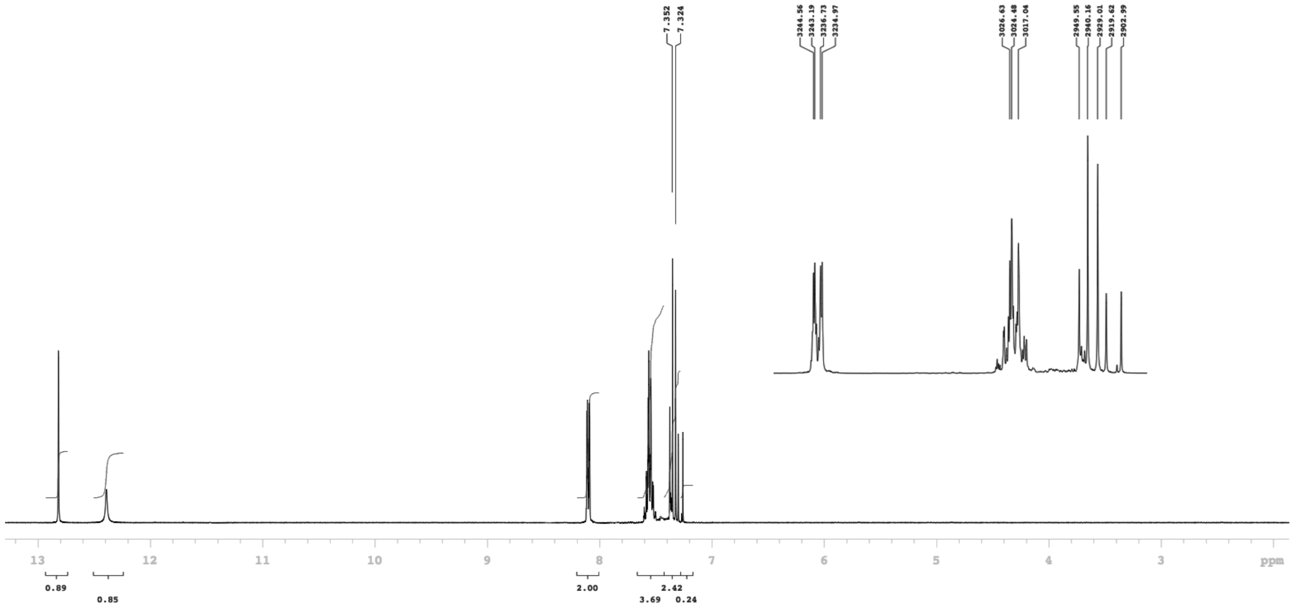
7.352
7.324
3244.56
3243.19
3236.73
3234.97
3026.63
3024.48
3017.04
2949.55
2940.16
2929.01
2919.62
2902.99
13 12 11 10 9 8 7 6 5 4 3 ppm
0.89
0.85
2.00
2.42
3.69
0.24

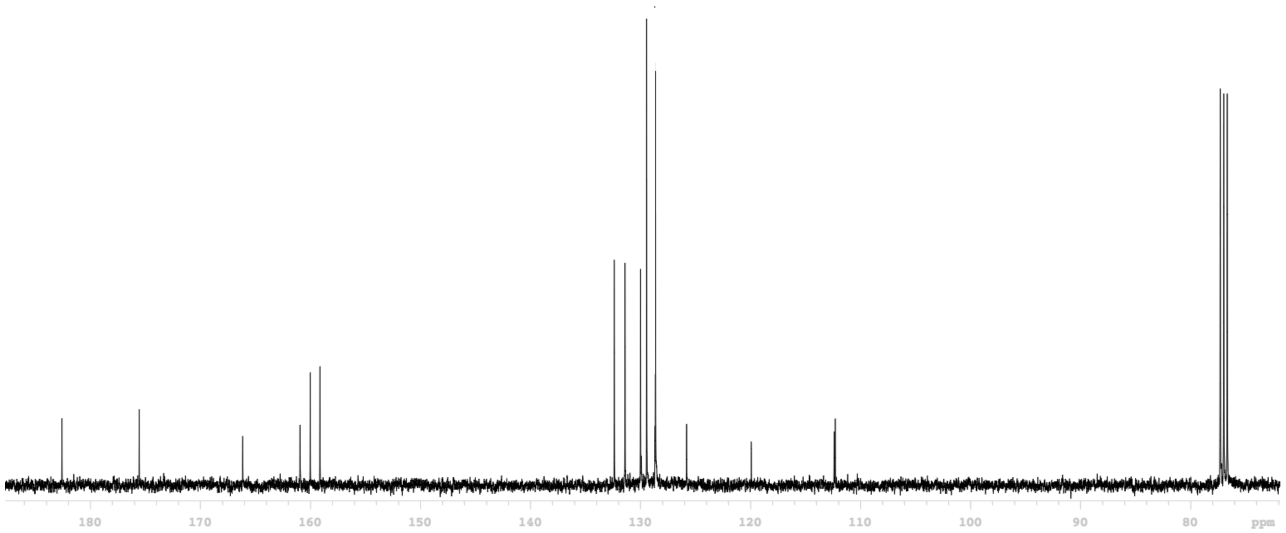
180 170 160 150 140 130 120 110 100 90 80 ppm

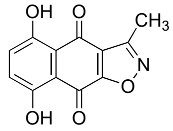
OH
O
CH_3
N
O
OH
O

3b

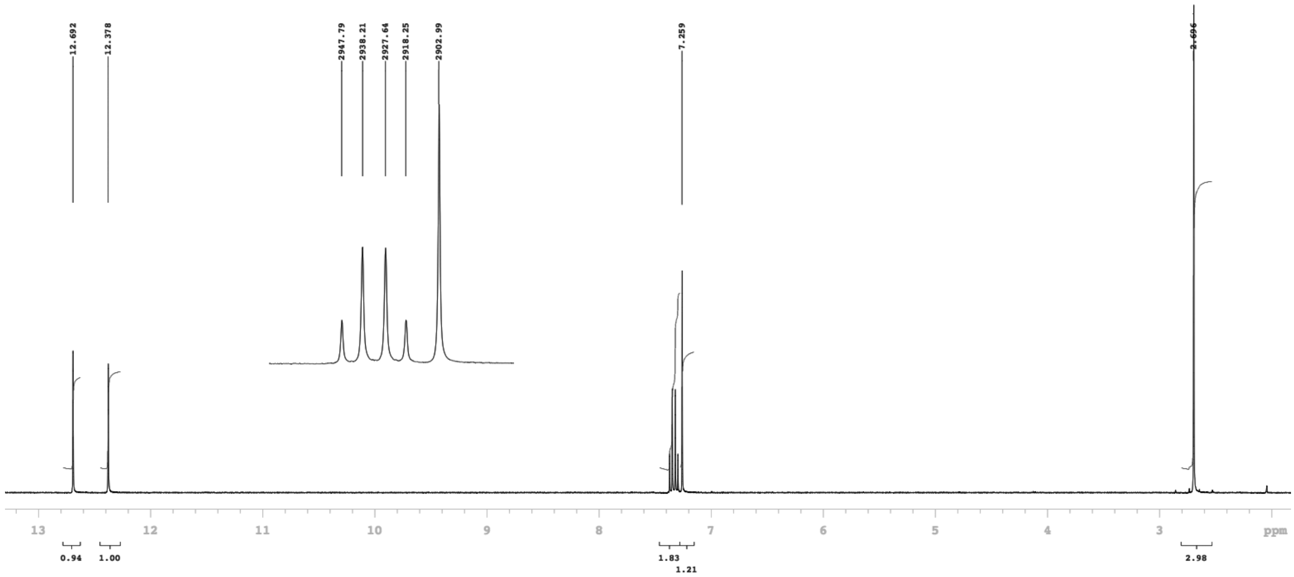
12.692
12.378
2947.79
2938.21
2927.64
2918.25
2902.99
7.259
2.696
13 12 11 10 9 8 7 6 5 4 3 ppm
0.94
1.00
1.83
1.21
2.98

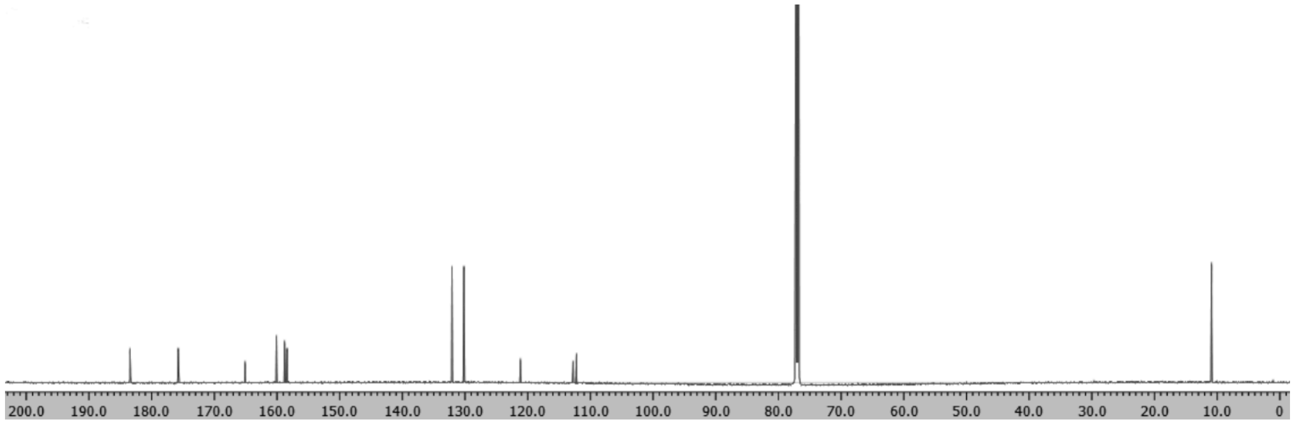
200.0 190.0 180.0 170.0 160.0 150.0 140.0 130.0 120.0 110.0 100.0 90.0 80.0 70.0 60.0 50.0 40.0 30.0 20.0 10.0 0

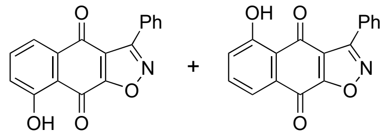

3c

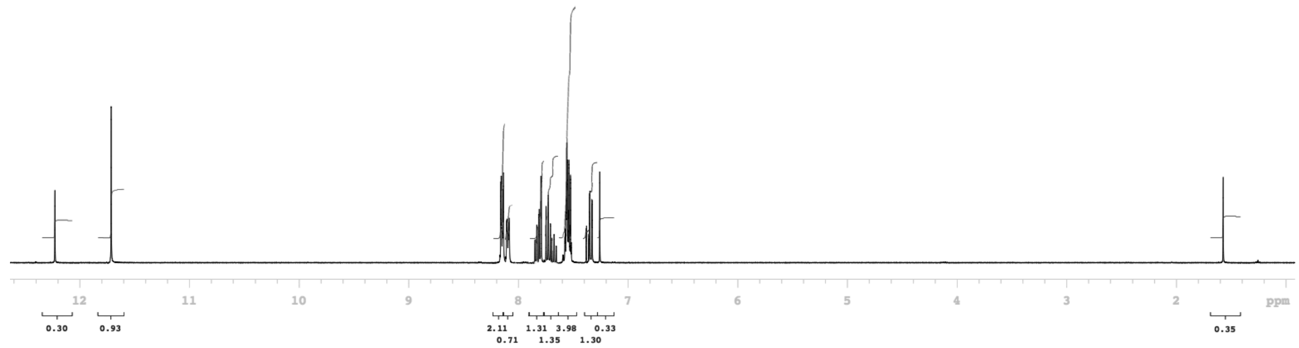

3c (major isomer)

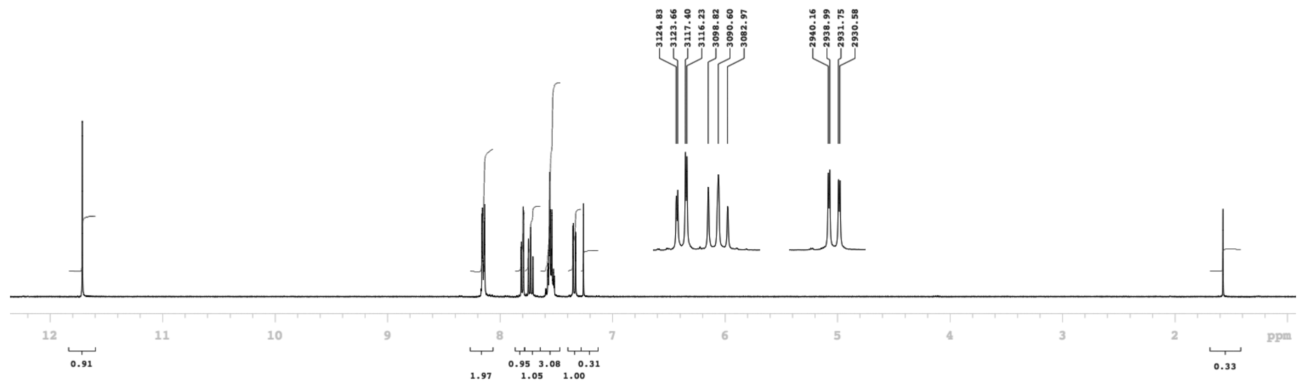

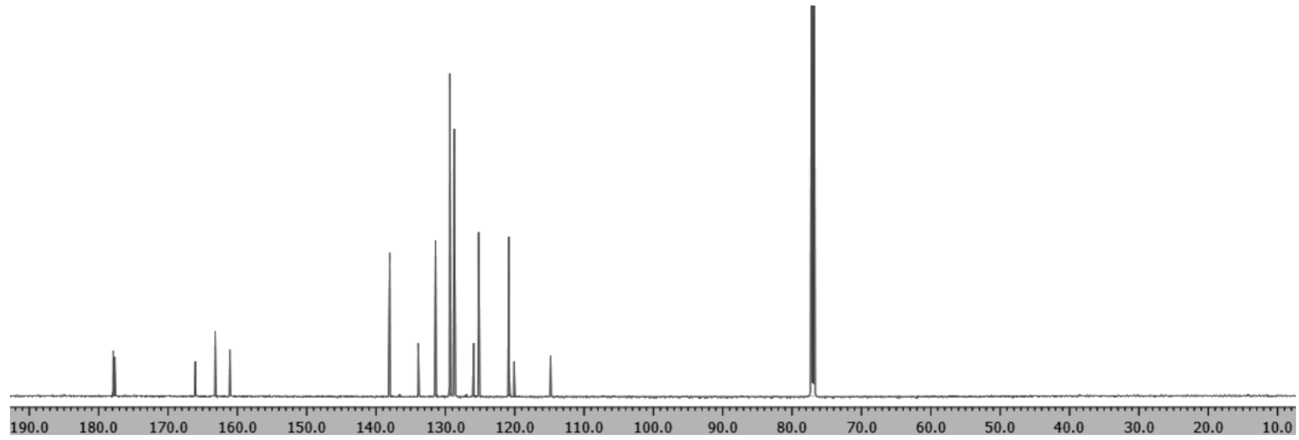

2. Primers used in this study (Table S1)

Primers	Sequence (5' – 3')	Reference
For cloning		
phoQ-NdeI-F	ggaattccatatggaacgcgaacgttacg	This study
phoQ-XhoI-R	aaccgctcgagttcatctttcggcgcag	This study
cpxA-F	ggagtcatatgaaaccggcgcgtaagctgaaaaacgctg	This study
cpxA-R	gtgatttggttgccgctgtataagcggagtctcgagtcgcat	This study
For RT-qPCR		
rt-rrn16S-2F	cgtgaagctggaatcgctagt	1
rt-rrn16S-2R	caaggcccgggaacgtat	1
rt-rpoB-F	gaccgggcggattgaca	1
rt-rpoB-R	gccatagtgggagtagtgaacgt	1
rt-yjeA-F	cattgccgcggacgat	1
rt-yjeA-R	caaagcggctgaaattttcttt	1
rt-yoeB-2F	cgctagagaacatttttggaacac	1
rt-yoeB-2R	tgatgggcaaactgctttttt	1

1. Fakhruzzaman Md, Inukai Y, Yanagida Y, Kino H, Igarashi M, Eguchi Y, et al. Study on in vivo effects of bacterial histidine kinase inhibitor, Waldiomycin, in *Bacillus subtilis* and *Staphylococcus aureus*. *J. Gen. Appl. Microbiol.*, 2015; 61: 177-184.

3. Histidine kinases (HKs) used in the autophosphorylation assays (Table S2)

HKs	Description	His-tag (His×6) location	Plasmids	References
WalK	*Bacillus subtilis* WalK(207-611)	N-terminal	pETBsWalKtrun	1
EvgS	*Escherichia coli* EvgS(559-1197) D1009A*	C-terminal	pETEcEvgS(559-1197)D1009A	2
EnvZ	*Escherichia coli* EnvZ(223-450)	C-terminal	pETEcEnvZ(223-450)	3
PhoQ	*Escherichia coli* PhoA(261-486)	C-terminal	pETEcPhoQ(261-486)	This study
CpxA	*Escherichia coli* CpxA(188-457)	C-terminal	pETEcCpxA(188-457)	This study

* D1009A mutant was used to prevent the phosphor-relay from the autophosphorylated His901 to D1009.

1. Okada A, Igarashi M, Okajima T, Kinoshita N, Umekita M, Sawa R, et al. Walkmycin B targets WalK (YycG), a histidine kinase essential for bacterial cell growth. *J. Antibiot. (Tokyo)* 2010; 63: 89-94.
2. Kinoshita-Kikuta E, Kinoshita E, Eguchi Y, Yanagihara S, Edahiro K, Inoye Y, et al. Functional characterization of the receiver domain for phophorelay control in hybrid histidine kinases. *PLoS ONE* 2015; 10: e0132598.
3. Eguchi Y, Okajima T, Tochio N, Inukai Y, Shimizu R, Ueda S, et al. Angucycline antibiotic waldiomycin recognizes common structural motif conserved in bacterial histidine kinases. *J. Antibiot. (Tokyo)* 2017; 70: 251-58.
